# Weight Management Interventions in Women with and without PCOS: A Systematic Review

**DOI:** 10.3390/nu9090996

**Published:** 2017-09-08

**Authors:** Josefin Kataoka, Eliza C. Tassone, Marie Misso, Anju E. Joham, Elisabet Stener-Victorin, Helena Teede, Lisa J. Moran

**Affiliations:** 1 Institute of Neuroscience and Physiology, Department of Physiology, Sahlgrenska academy, University of Gothenburg, Box 430, 405 30 Gothenburg, Sweden; Josefin.Kataoka@vgregion.se; 2 Department of Obstetrics and Gyneacology, NU Hospital Groups, Lärketorpsvägen 4, 461 73 Trollhättan, Sweden; 3 Monash Centre for Health Research and Implementation, School of Public Health and Preventive Medicine, Monash University, Locked Bag 29, Clayton, VIC 3168, Australia; eliza.tassone@monash.edu (E.C.T.); marie.misso@monash.edu (M.M.); Anju.Joham@monash.edu (A.E.J.); Helena.Teede@monash.edu (H.T.); 4 Department of Physiology and Pharmacology, Karolinska Institutet, 171 77 Stockholm, Sweden; elisabet.stener-victorin@ki.se; 5 Monash Partners Academic Health Sciences Centre, Locked Bag 29, Clayton, VIC 3168, Australia; 6 Robinson Research Institute, School of Paediatrics and Reproductive Health, University of Adelaide, 55 King William Street, North Adelaide, SA 5006, Australia

**Keywords:** polycystic ovary syndrome, obesity, insulin resistance, weight loss, systematic review

## Abstract

Polycystic ovary syndrome (PCOS) is a common endocrinopathy among women associated with reproductive, metabolic and psychological features. While weight management is recommended as first-line treatment, it is unclear if women with PCOS achieve similar benefits as women without PCOS. This systematic review thus aimed to compare the efficacy of weight management interventions in women with and without PCOS. Databases were searched until May 2017. The primary outcome was weight and anthropometric, reproductive, metabolic and psychological measures were secondary outcomes. Of 3264 articles identified, 14 studies involving *n* = 933 (*n* = 9 high and *n* = 5 moderate risk of bias) met the inclusion criteria. No statistically significant differences in weight or weight loss following the intervention were found between women with and without PCOS in five studies, with the remaining studies not comparing the difference in weight or weight loss between these groups. Secondary outcomes did not differ significantly between the two groups. This review identified that there is a paucity of high quality research in this area and that more rigorous research is needed.

## 1. Introduction

Polycystic ovary syndrome (PCOS) is one of the most common endocrinopathies in women of reproductive age, with a prevalence of 6–18%, depending on the diagnostic criteria used and the population studied [1,2,3]. The condition is underpinned by insulin resistance (IR) and hyperandrogenism. These hormonal abnormalities both contribute to clinical features including hirsutism or acne, oligo-/anovulation and polycystic ovarian morphology, type 2 diabetes (DM2), metabolic syndrome and cardiovascular disease [4,5,6,7]. PCOS also accounts for the majority of cases of anovulatory infertility [8], and women with PCOS have an increased risk of pregnancy and neonatal complications [9]. Women with PCOS also have a higher risk of psychological complications, including depression and anxiety [10].

The etiology of PCOS is complex and poorly understood. Both genetic and environment factors contribute to the syndrome [11], and both gonadotropin hypersecretion and IR increase androgen secretion [12,13,14]. Women with PCOS appear to have a higher rate of weight gain and a higher prevalence of overweight, obesity and central obesity, compared to women without PCOS [15,16,17]. Obesity, especially central obesity, worsens the clinical and biochemical presentation of the syndrome, contributing to IR, hyperandrogenism, reproductive disorders, diabetes and cardiovascular disease [18,19,20]. Weight loss, in turn, improves all the features of PCOS, and lifestyle (diet, physical activity and behavior) changes and weight management are recommended as first line treatment for PCOS [21,22] to improve hormonal disturbances and to prevent future reproductive and metabolic complications. Preconception lifestyle interventions and weight loss are also recommended before infertility treatment is initiated [23], and lead to higher ovulation rates compared to oral contraceptive pretreatment [24].

It has been proposed that weight management interventions may be less effective in women with PCOS compared to those without PCOS given the higher rate of longitudinal weight gain in PCOS [17]. This may be related to the hormonal aberrations of PCOS such as hyperandrogenemia or IR, contributing to abnormalities in energy homeostasis and dietary intake including gut hormone regulation [25,26], or an altered metabolism due to reduced postprandial thermogenesis [27]. However, the literature is limited and contradictory. The aim of this systematic review is thus to assess the effect of lifestyle (dietary and non-dietary) weight management interventions on anthropometric, reproductive and metabolic outcomes in women with PCOS, compared to women without PCOS.

## 2. Materials and Methods 

### 2.1. Selection Criteria

This systematic review was conducted in accordance with the Preferred Reporting Items for Systematic Reviews and Meta-Analyses (PRISMA) Statement checklist [28]. The detailed PICO (Population, Intervention, Comparison, Outcome) framework in Supplementary Table S1, established a priori, was used to include and exclude articles for this systematic review. Briefly, articles were included if they reported a study that compared women with PCOS to women without PCOS; following a weight management intervention; and investigated anthropometric, fertility, reproductive non-fertility, metabolic, quality of life or emotional wellbeing outcomes. The primary outcome was weight management defined as either weight loss, weight maintenance or prevention of weight gain. Secondary anthropometric, reproductive, metabolic and psychological outcomes are listed in Table S1 and were analysed based on the most complete data, which were body mass index (BMI), waist circumference for fat distribution, computed tomography for fat and lean mass, number of ovulations, total testosterone, sex hormone-binding globulin (SHBG), free androgen index (FAI), fasting glucose, oral glucose tolerance test (OGTT), fasting insulin, total, low-density lipoprotein (LDL) and high-density lipoprotein (HDL) cholesterol, triglycerides, and blood pressure.

### 2.2. Systematic Search for Evidence 

A systematic search (Supplementary Table S2) was developed incorporating terms related to weight management (including lifestyle, behaviour, pharmacological, surgical and complementary and alternative interventions) and combined with terms related to PCOS. The search strategy was limited to English language articles and there were no limits on year of publication. The following electronic databases were searched via the OVID platform to identify relevant literature up to May 2017: Ovid MEDLINE(R) 1946 to Present with Daily Update; Ovid MEDLINE(R) In-Process and Other Non-Indexed Citations; Evidence-Based Medicine (EBM) Reviews incorporating Cochrane Database of Systematic Reviews, EBM Reviews—ACP Journal Club, EBM Reviews—Database of Abstracts of Reviews of Effects, EBM Reviews—Cochrane Central Register of Controlled Trials, EBM Reviews—Cochrane Methodology Register, EBM Reviews—Health Technology Assessment, EBM Reviews—National Health Service (NHS) Economic Evaluation Database; PsycINFO; and EMBASE. CINAHL Plus was searched separately on the same date. Bibliographies of relevant articles were also searched for identification of additional studies.

### 2.3. Inclusion of Studies

To determine the literature to be assessed further, one trained reviewer (J.K. for search to 23 September 2015 and E.C.T for search to 11 May 2017) screened the titles, abstract sections and keywords of every article retrieved by the search strategy using the selection criteria described in Table S1. Full articles were retrieved for further assessment if the information given suggested that the article met the selection criteria, or if it was unclear. Three additional reviewers were consulted throughout the screening process.

### 2.4. Quality Appraisal of the Evidence 

Methodological quality, in terms of risk of bias, of the included studies was assessed by two reviewers (J.K. and E.C.T.) in consultation with experienced reviewers (M.M. and L.J.M.) using criteria developed a priori [29], designed for cohort studies. Individual quality items were investigated using a descriptive component approach that assessed selection bias, performance bias, attrition bias, reporting bias, potential confounding, and appropriateness of statistical analysis. Any disagreement or uncertainty was resolved by discussion among authors to reach a consensus. Using this approach, each study was allocated a risk of bias rating. Where there was more than one article describing a study, all articles were used to complete one risk of bias assessment on the study.

### 2.5. Data Extraction

Double data extraction, according to the selection criteria described in Table S1 was conducted by two independent reviewers for the original search (J.K. and A.J.) and by two independent reviewers for the updated search (A.J. and E.C.T.). Information was collected on relevant outcome data and included point estimates, measures of variability and number of participants. Where there was more than one article describing a study, data from the most current and comprehensive article was extracted, and any additional outcome data reported in additional articles were subsequently extracted.

### 2.6. Data Synthesis

Due to the heterogeneity in interventions, a meta-analysis was not performed, results are thus presented narratively and in tabular form. Publication bias could not be assessed due to the lack of meta-analysis as this requires a meta-analysis to be performed on 10 or more studies. All studies reported baseline and endpoint values, except for two studies that reported the difference from baseline to post-intervention. Outcome data has been excluded in instances where only the mean was presented with no corresponding variance data (standard deviation (SD), standard error (SE), or confidence interval (CI)). Where necessary, unit conversions were performed so that results are presented in SI units; in some instances, where conversion factors could not be obtained, the unit from the original manuscript has been retained.

## 3. Results

**Figure 1:**
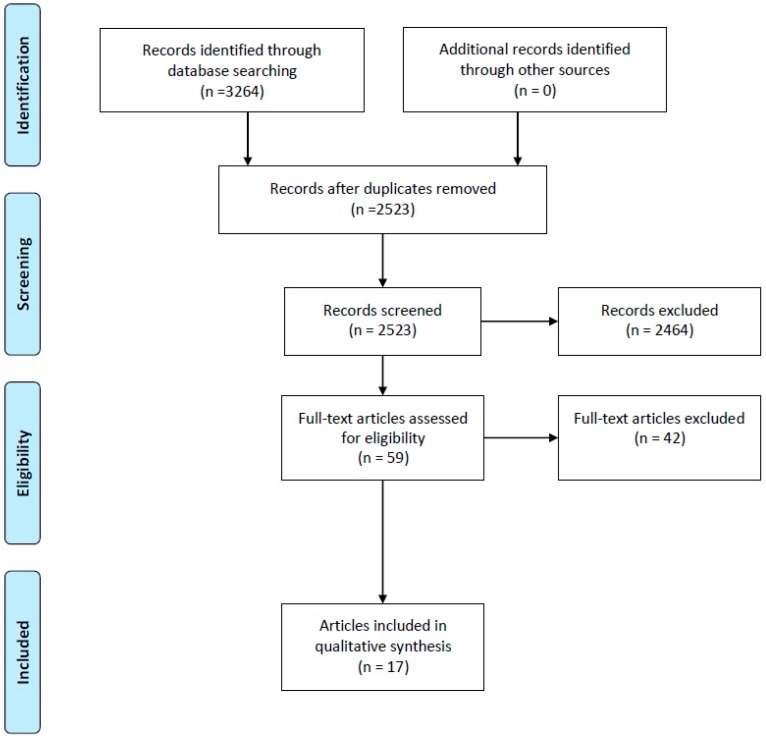
Preferred Reporting Items for Systematic Reviews and Meta-Analyses (PRISMA) flow diagram.

The search returned 3264 articles (Figure 1). Fifty-nine full text articles were retrieved for further evaluation and 17 articles [30,31,32,33,34,35,36,37,38,39,40,41,42,43,44,45,46] reporting 14 studies met the inclusion criteria. Of these 17 articles, one study was reported across two articles [31,32] and one study [35] reported data across different time points [36,37]. Two studies reported data from two intervention groups, respectively [38,39]. A table of studies, excluded based on full text, is found in Supplementary Table S3: Table of Excluded Studies. All between group comparisons presented here are presented as the authors reported that data and is per protocol for all analyses.

### 3.1. Characteristics and Quality of Included Studies

The characteristics of included studies are reported in Table 1. All studies were comparative/parallel design. Two were designed as randomized controlled studies (RCTs) but comprised two intervention groups, respectively, and were used as comparative studies [38,39]. Five studies had a moderate risk of bias, whilst nine studies had a high risk of bias (Supplementary Table S4). Interventions varied across the included studies (diet, diet + behavior change program, diet + Metformin, diet + anti-obesity drug, anti-obesity drug, diet + anti-obesity drug + exercise, bariatric surgery, and various exercise training programs), as did the duration of interventions. Nine studies were designed with the specific aim of weight loss [34,37,38,39,41,42,43,45,46], while the remaining five studies did not state if the aim was weight loss [30,31,33,40,44]. The sample size of the studies varied from 31 to 1016.

### 3.2. Outcomes: Anthropometric 

#### 3.2.1. Weight

Six studies including eight intervention groups reported weight as endpoint data [31,38,39,42,45,46], whilst a further three studies reported change in weight [34,41,43], and one study reported the estimated difference in weight from baseline to post-intervention [44] (Table 2). Five of the studies reported that the difference in weight post-intervention or the change in weight from baseline between the PCOS and non-PCOS groups was not statistically significantly different, whilst the remainder of studies did not report a *p*-value.

#### 3.2.2. Body Mass Index (BMI)

Thirteen intervention groups from 12 studies [30,31,33,36,38,40,41,42,43,44,45,46] reported BMI (Table 2). One study reported a statistically significant difference in BMI following bariatric surgery (*p* = 0.013), with women with PCOS having a lower BMI post-surgery than women without PCOS [45]. This study, however, had a high risk of bias, with a number of key elements of the study protocol not reported and should therefore be interpreted with caution. Three studies reported a non-statistically significant difference in BMI between the two groups, whilst eight studies did not report a *p*-value.

#### 3.2.3. Waist Circumference (WC)

Seven studies including nine intervention groups [31,33,37,38,39,44,46] reported WC (Table 2). The majority of studies did not compare between-group differences after the interventions, with only one study reporting a non-statistically significant difference in WC between groups following progressive resistance training [44].

#### 3.2.4. Body Composition 

Three intervention groups from two studies [31,38] measured body composition with computed tomography (CT). One study with an exercise intervention reported abdominal visceral fat (VF) and subcutaneous fat (SCFAT). Two intervention groups from one study with the interventions diet + Metformin and diet + placebo reported visceral adipose tissue area (VAT) and subcutaneous adipose tissue area (SAT). None of the studies reported whether there was between-group statistical significance (Table 2).

### 3.3. Outcomes: Fertility

#### Ovulation 

One study with a diet intervention [34] reported double the number of ovulatory events in women without PCOS compared to women with PCOS, which was statistically significant (Table 3).

### 3.4. Outcomes: Reproductive Non-Fertility

#### 3.4.1. Total Testosterone

Eight intervention groups from seven studies reported total testosterone [30,31,33,35,38,40,44]. One study with a hypocaloric diet + an anti-obesity drug intervention (Orlistat) [30], and another study with an anti-obesity drug intervention (Orlistat) + a hypocaloric diet + exercise intervention [35] reported a statistically significant difference in total testosterone following intervention between the two study groups, with the non-PCOS group having lower values in both studies (Table 3). However, as expected in both studies, the women with PCOS had statistically significantly higher levels of testosterone at baseline than women without PCOS.

#### 3.4.2. Sex Hormone-Binding Globulin (SHBG) 

Eight intervention groups from seven studies reported SHBG [30,31,33,35,38,40,44], with the majority of studies not reporting whether there were between-group statistical significance (Table 3). One study with a hypocaloric diet + anti-obesity drug (Orlistat) intervention [30] reported a statistically significant difference between groups (*p* < 0.05) following the intervention; however, there was also a statistically significant difference in SHBG at baseline between the groups (*p* < 0.05).

#### 3.4.3. Free Androgen Index (FAI) 

Five studies reported FAI [31,33,35,40,44], with one study with an anti-obesity drug intervention (Orlistat) + a hypocaloric diet + exercise intervention [35] reporting a statistically significant difference in values post intervention between groups (*p* = 0.021), with women in the non-PCOS group having a lower mean value (Table 3). However, as expected, women with PCOS in this study had a higher mean FAI at baseline (*p* < 0.001).

### 3.5. Outcomes: Metabolic

#### 3.5.1. Fasting Glucose 

Eleven intervention groups from nine studies reported fasting glucose [30,31,35,38,39,40,43,44,46]. The majority of studies did not report whether there were between-group statistical significance, and three studies [35,43,44] reported that there was no statistically significant difference in fasting glucose post-intervention between the two groups (Table 4).

#### 3.5.2. Oral Glucose Tolerance Test (OGTT-Glucose)

Three intervention groups from two studies reported OGTT-glucose [30,39]. None of these studies reported whether there were between-group statistical significance following the interventions (Table 4).

#### 3.5.3. Fasting Insulin 

Eleven intervention groups from nine studies reported fasting insulin [30,31,33,35,38,39,40,44,46]. Only one of these studies reported a *p*-value for the between-group difference following the interventions (*p* = 0.58) [44] (Table 4).

#### 3.5.4. Lipids

Four intervention groups from three studies reported results for blood lipids [31,35,39]. One study with an anti-obesity drug intervention (Orlistat) + a hypocaloric diet + exercise intervention [35] reported a statistically significant difference in total, LDL and HDL cholesterol post-intervention between groups, with women in the PCOS group having lower mean values (Table 5).

#### 3.5.5. Blood Pressure (BP)

Three intervention groups from two studies reported results for BP [32,39]. None of the studies reported whether there were between-group statistical significance (Table 5).

## 4. Discussion

In this systematic review, we evaluated for the first time the effect of weight management interventions in women with PCOS compared to women without PCOS. We identified 14 studies in 933 women with considerable clinical heterogeneity across a range of lifestyle (dietary and non-dietary) and non-lifestyle weight management interventions including diet, diet + behavior change program, diet + Metformin, diet + anti-obesity drug, anti-obesity drug, diet + anti-obesity drug + exercise, bariatric surgery, and various exercise training programs. Overall, there were no statistically significant differences in weight at the end of an intervention or in change in weight, between groups for five of the interventions included, with the remainder of studies not reporting whether there was a difference between groups post-intervention. For the majority of secondary endpoints, there was little difference between groups. Furthermore, five of the included studies had a moderate risk of bias, whilst nine studies had a high risk of bias.

The included studies are set in a research environment, with structured interventions, isocaloric intake and frequent contact and interaction with trained professionals. This mirrors many structured lifestyle interventions, and like all research in lifestyle interventions, occurred in a motivated research population.

As with all lifestyle intervention studies, our results may not reflect the outcomes of community-based studies or self-induced lifestyle change. Our previous research has shown a greater 10-year weight gain (2.6 kg (95% CI: 1.2–4.0)) in community-based cohort studies [17], and an elevated prevalence of overweight (risk ratio (RR) (95% CI): 1.95 (1.52, 2.50)), obesity (2.77 (1.88, 4.10)), central obesity (1.73 (1.31, 2.30)) and BMI (2.5 kg/m^2^ (95% CI: 1.9–3.1)) in systematic reviews [15] of women with PCOS compared to women without PCOS. This may relate to hyperinsulinemia, reduced postprandial thermogenesis [27], altered metabolic rate [47], impaired regulation of gut hormones or appetite regulation or limited self-regulation of food intake [48,49,50]. While the evidence here is not reliable enough to state conclusively if there is no difference in the effect of weight management interventions in women with and without PCOS, if this was indeed the case, this could indicate that appetite and self-regulation may be more prominent contributors to weight in PCOS. These mechanisms are more likely to selectively manifest in a free-living environment with ad libitum food intake. Our past longitudinal community cohort studies also show unselected community dwelling women with PCOS report a higher caloric intake, which corresponds closely to higher rates of weight gain [51]. More research into appetite and self-regulation around food intake are needed in PCOS.

The majority of secondary endpoints were similar between women with and without PCOS following the specified interventions. Similar to weight, a large number of the included studies did not report whether there was a statistically significant difference between groups for secondary endpoints following the intervention. While for a limited number of outcomes, there was a statistically significant difference in these measures at study completion, this difference was also present at baseline indicating that examining the within-group change from baseline to the end of the intervention would be more appropriate. We also report changes in secondary outcomes including total testosterone and SHBG at study completion between women with and without PCOS. However, women with PCOS generally have hyperandrogenism and lower SHBG [52] and differences at the end of the intervention are again likely related to baseline differences.

Here, it was not possible to determine the relative effectiveness of specific dietary or non-dietary interventions. Based on our findings reported elsewhere [21,22], recommendations in PCOS for lifestyle or pharmacological intervention and weight management should mirror those for the general population. These include a diet with reduced energy intake and a weight loss maintenance program for maintaining weight. Pharmacological treatment can increase efficacy of lifestyle interventions [53,54]. A recent meta-analysis reported that a metformin plus lifestyle intervention results in more reduction in fat and improved menstrual cyclicity, compared to lifestyle alone in PCOS [53]. In general populations, a large meta-analysis (*n* = 80 studies, *n* = 26,455 participants) reported that diet interventions in isolation are as effective for short-term (six months) weight loss compared to anti-obesity drugs. Long-term (24 months) weight-loss, however, was greater with anti-obesity drugs [55]. Overall, bariatric surgery is an option for those who have not responded to these interventions with sufficient weight loss or for those with morbid obesity and co-morbidities [56,57]. Overall, the relative efficacy of different weight management interventions in women with and without PCOS warrants further investigation.

The strengths of this study include the comprehensive range of lifestyle and weight management interventions and of obesity-related outcomes studied. We also included studies where weight loss was not the specific goal, broadening applicability of our findings to weight gain prevention, weight maintenance and use in lean women with PCOS. The weaknesses of this review include the inability to statistically combine study results due to clinical heterogeneity. Additionally, this systematic review was limited to studies in English. Many of the included studies did not report numeric differences between groups and relevant data could not be extracted. The identified studies also had moderate to high risk of bias and were generally small with significant drop-outs. Many did not strictly exclude PCOS status in their controls. Future research should address these methodological weaknesses to improve study quality. We also did not prospectively register this review on PROSPERO; however, it was designed in 2012 at which time PROSPERO had only been active for a relatively short amount of time, and it was not yet standard practice for all systematic reviews to be prospectively registered. Additional biases may also have occurred through identifying articles only in English and through not contacting authors for missing data. Detailed reporting of inclusion and exclusion criteria, increased participant numbers, attrition rates, anthropometric measurement protocols, and publishing study protocols are suggested to improve the reliability and validity of future research. Endpoint reporting could also be improved across the range of obesity-related health implications in PCOS, and through the use of a pre-specified list of outcomes similar to that suggested for infertility treatment [58].

## 5. Conclusions

We report on the first systematic review comparing the effectiveness of weight management strategies in women with and without PCOS. Our findings are that there is insufficient evidence to indicate significant differences in weight loss in women with PCOS compared to women without PCOS with lifestyle or weight management strategies. We cannot determine the relative efficacy of different types of weight management interventions and note considerable limitations with the current studies. Further research with larger samples is warranted in controlled clinical trials and free-living environments assessing a full range of defined PCOS and obesity-related health implications. At the current time, recommendations for weight management for the general population should be applied for women with PCOS with some degree of confidence that similar efficacy of structured interventions is likely.

## Figures and Tables

**Table 1:** Characteristics of included studies.

Study	Design	Country	PCOS Details at Baseline N (Completers)	Non-PCOS Details at Baseline N (Completers)	Attrition Rate	Current Medication	Specific Exclusion Criteria	Intervention	Outcomes	Risk of Bias
Diamanti-Kandarakis 2007 [30]	Comparative study	Greece	Age 27.5 ± 5.77 BMI 35.4 ± 5.31 ESHRE/ASRM *n* = 29	Age 32.1 ± 5.64 BMI 36.4 ± 6.47 *n* = 18	Not reported	Not reported	Galactorrhea	Duration: 24 weeks Normal protein diet, 600 kcal/day energy deficit, Orlistat (Xenical^®^, Roche) 120 mg × 3 times daily Aim of weight loss: not stated	Anthropo-metric: BMI Reproductive: T, SHBG Metabolic: FG, 2HRG, FI	Moderate
Moran 2007 [34]	Comparative study	Australia	Age 31.7 ± 6.2 BMI 35.7 ± 5.8 ESHRE/ASRM *n* = 15	Age 37.1 ± 4.7 BMI 35.5 ± 5.1 *n* = 17	PCOS 17% Non-PCOS 11%	No hormonal or insulin-sensitising drugs pre study	Pregnancy, breastfeeding	Duration: 8 weeks Energy restricted diet (two meals daily replaced with commercially available meal replacements) Aim of weight loss: Yes	Anthropo-metric: Weight, WC Reproductive: T, SHBG, FAI Metabolic: FI, lipids Others: IL-6, TNF-α	Moderate
Hutchison 2011 [31] Harrison 2012 [32]	Comparative study	Australia	BMI > 27 (37.4 ± 1.5) Age 20–40 (29.5 ± 1.4) NIH *n* = 13	BMI > 27 (35.7 ± 1.3) Age 20-40 (35.0 ± 1.1) *n* = 8	PCOS 35% Non-PCOS 43%	No hormonal or insulin-sensitising drugs pre study	Diabetes, adrenal disorders, recent weight change, regular physical activity, pregnancy, breastfeeding, smoking	Duration: 12 weeks supervised intensified exercise training 60 min three times weekly Aim of weight loss: not stated	Anthropo-metric: WC, Weight, BMI, VF, SCFAT Reproductive: T, SHBG, FAI Metabolic: FG, FI, lipids, BP	Moderate
Cheang 2016 [43]	Comparative study	USA	Age 26.9 ± 4.6 BMI 36.6 ± 5.1 Modified ESHRE/ASRM *n* = 16	Age 27.5 ± 5.7 BMI 35.8 ± 4.8 *n* = 15	PCOS 53% Non-PCOS 44%	No unstable medication use for 6 months for disorders such as hypertension or dyslipidemia	Weight loss attempts in 3 months pre study, diabetes, pulmonary, cardiac, renal, neurologic, hepatic, psychiatric, infectious, neoplastic, malignant disease, pregnancy Non-PCOS: history gestational diabetes, family history abnormal glucose tolerance, hypertension, dyslipidemia	Duration: 8 weeks Standardized hypocaloric diet (50% carbohydrate, 20% protein, 30% fat) with 500–1000 kcal/day deficit. No modification of physical activity or other weight loss methods. Aim of weight loss: Yes	Anthropometric: Weight, BMI Metabolic: FG, FI	Moderate
Kogure 2016 [44]	Comparative study	Brazil	Age 28.1 ± 5.4 BMI 28.4 ± 6.0 ESHRE/ASRM *n* = 45	Age 29.6 ± 5.2 BMI 26.2 ± 5.7 *n* = 52	PCOS 38% Non-PCOS 46%	No hormonal contraceptive us drugs pre or during study	Systemic diseases, smoking, pregnancy	Duration: 4 months Progressive resistance training (PRT) for 1 h/day three times per week. Aim of weight loss: not stated	Anthropometric: Weight, BMI, WC, TFFM, % body fat Reproductive: T, SHBG, FAI Metabolic: FG, FI, HOMA-IR	Moderate
Villa 1999 [40]	Comparative study	Italy	Age 26.3 ± 5 BMI 27.5 ± 6.8 NIH *n* = 22	Age not reported BMI 27.4 ± 6.8 *n* = 14	Not reported	Not reported	Not reported	Duration: 4–5 weeks 50 mg Naltrexone daily Aim of weight loss: not stated	Anthropo-metric: BMI Reproductive: T, SHBG, FAI Metabolic: FG, FI	High
Kowalska 2001 [33]	Comparative study	Poland	Age 25.3 ± 4.8 BMI 34.7 ± 6.0 NIH *n* = 11	Age 27.9 ± 7.3 BMI 36.2 ± 6.0 *n* = 19	PCOS 27% Non-PCOS 40%	Not reported	No additional	Duration: 4–5 months Hypocaloric diet (1200–1400 kcal/day), Metformin 500 mg three times daily Aim of weight loss: not stated	Anthropo-metric: BMI, WC Reproductive: T, FAI, SHBG Metabolic: FI	High
Panidis 2014 [35] Vosnakis 2013 [36] Panidis 2008 [37]	Comparative study	Greece	Age 26.1 ± 6.4 BMI 34.5 ± 5.9 ESHRE/ASRM *n* = 101	Age 31.5 ± 4.7 BMI 34.9 ± 5.4 *n* = 29	Not reported	No hormonal or insulin-sensitising drugs pre or during study	Galactorrea	Duration: 6 months Normal protein, energy-restricted diet (600 kcal/day energy deficit, moderate intensity aerobic exercise, 1 h × 3 times/week, Orlistat 120 mg before each meal) Aim of weight loss: Yes	Anthropo-metric: WC, BMI Reproductive: T, FAI, SHBG Metabolic: FG, lipids	High
Kahal 2015 [41]	Comparative study	UK	Age 33.9 ± 6.7 BMI 37.9 ± 5.0 ESHRE/ASRM *n* = 13	Age 33.5 ± 7.1 BMI 36.5 ± 4.6 *n* = 12	PCOS 32% Non-PCOS 29%	No medication	Alcohol intake >14 units/week Non-PCOS: history of hirsutism or menstrual irregularities	Duration: 6 months Liraglutide 0.6 mg o.d subcutaneous injection for 1 week, 1.2 mg o.d for 1 week, and 1.8 mg o.d thereafter for 6 months. No diet or exercise advice given. Aim of weight loss: Yes	Anthropometric: Weight, BMI	High
Nikokavora 2015 [42]	Comparative study	UK	Age 35.7 ± 8.9 BMI 40.0 ± 6.3 Diagnostic criteria not reported *n* = 137	Age 35.8 ± 8.9 BMI 40.0 ± 6.3 *n* = 137	PCOS 73% Non-PCOS 73%	Not reported	Type 1 diabetes, porphyria, lactose intolerance, major cardio- or cerebrovascular disease, history of renal or hepatic disease, cancer, epilepsy, major psychological or eating disorders, breastfeeding, pregnant, birth or miscarriage prior 3 months	Duration: 12 weeks Commercial weight management program (LighterLife Total), 600 kcal (36% protein, 36% carbohydrate, 28% fat) as food packs alongside behavior change program Aim of weight loss: Yes	Anthropometric: Weight, BMI	High
Bhandari 2016 [45]	Comparative study	India	Age 27.8 ± 4.50 BMI 42.5 ± 5.71 ESHRE/ASRM *n* = 43	Age 29.3 ± 4.96 BMI 45.0 ± 6.11 *n* = 32	Not reported	No hormonal, fertility or insulin-sensitising drugs pre or during study	Systemic diseases like hypothyroidism or hyperprolactinaemia, surgical complications intra or post operatively	Duration: 6 months post-surgery Sleeve gastrectomy (bariatric surgery) Aim of weight loss: Yes	Anthropometric: Weight, BMI Other: Abnormal menstrual cycles	High
Al-Eisa 2017 [46]	Comparative study	Egypt	Age 27.9 ± 4.1 BMI 33.5 ± 2.75 ESHRE/ASRM *n* = 30	Age 27.6 ± 5.7 BMI 31.7 ± 3.8 *n* = 30	Not reported	No hormonal drugs pre or during study	Normal BMI, other diseases such as diabetes or viral infections	Duration: 12 weeks treadmill walking, 45 min three times per week for 12 weeks. Aim of weight loss: Yes	Anthropometric: Weight, BMI, WC Metabolic: FG, FI	High
Pasquali 2000 [38]	RCT	Italy	BMI > 28 WHR > 0.80 NIH *n* = 18	BMI > 28 WHR > 0.80 *n* = 17	Diet: PCOS 0% Non-PCOS 25% Metform-in: PCOS 17% Non-PCOS 0%	No hormonal or insulin-sensitising drugs pre study	Diabetes, renal or liver dysfunction	Duration: 6 months One month hypocaloric diet (1200–1400 kcal daily), Metformin 850 mg twice daily. Aim of weight loss: Yes	Anthropo-metric: Weight, BMI, SAT, VAT Reproductive: T, SHBG Metabolic: FG, FI	Moderate
Toscani 2011 [39]	RCT	Brazil	Age 22.7 + 5.68 Most participants BMI ≥ 25 NIH *n* = 18	Age 29.4 + 5.74 Most participants BMI ≥ 25 *n* = 22	Not reported	No hormonal drugs pre study	Diabetes, renal dysfunction	Duration: 2 months Diet 1: HP (30% protein, 40% carbohydrate, 30% fat) Diet 2: NP (15% protein, 55% carbohydrate, 30% fat) Aim of weight loss: Yes	Anthropo-metric: Weight, WC, BMI Reproductive: T, SHBG, FAI Metabolic: BP, FG, 2HRG, FI, 2HRI, lipids	High

2HRG: 2 h glucose; 2HRI: 2 h insulin; BMI, body mass index; BMR, basal metabolic rate; BP, blood pressure; FAI, free androgen index; FG, fasting glucose; FI, fasting insulin; IL-6, interleukin-6; PCOS, polycystic ovary syndrome; SAT, subcutaneous abdominal tissue; SCFAT, subcutaneous fat; SHBG, sexual-hormone binding globulin; T, total testosterone; TFFM, total fat free mass; TFM, total fat mass; TNF-α, tumor necrosis factor-α; VAT, visceral abdominal tissue; VF, visceral fat; WC, waist circumference.

**Table 2:** Anthropometric outcomes.

Outcome	Reference	Intervention	Baseline PCOS: Mean ± SD	Baseline Non-PCOS: Mean ± SD	*p*-Value *	Post-Intervention PCOS: Mean ± SD	Post-Intervention Non-PCOS: Mean ± SD	*p*-Value *
**Weight (kg)**	Pasquali 2000 [38]	Hypocaloric diet	102 ± 19	106 ± 13	NR	97 ± 18	100 ± 13	NR
	Cheang 2016 [43]	Hypocaloric diet	99.2 ± 13.3	97.6 ± 15.4	0.7508	−4.08 ± 3.65	−4.69 ± 2.98	0.6281
	Moran 2007 [34]	Hypocaloric diet	95.1 ± 19.3	95.5 ± 16.5	NS	−3.9 ± 3.6	−4.5 ± 4.1	0.642
	Toscani 2011 [39]	High protein diet	74.62 ± 18.8	75.89 ± 13.49	NR	71.4 ± 15.45	74.54 ± 13.71	NR
	Toscani 2011 [39]	Normal protein diet	82.85 ± 15.18	77.51 ± 13.31	NR	79.82 ± 16.51	74.31 ± 13.88	NR
	Nikokavoura 2015 [42]	VLCD + behaviour change	108.3 ± 18.1	107.4 ± 19.8	0.713	89.8 ± 16.7	88.0 ± 17.6	0.19
	Pasquali 2000 [38]	Hypocaloric diet + Metformin	103 ± 18	101 ± 8	NR	94 ± 17	88 ± 7	NR
	Kahal 2015 [41]	AO drug (Liraglutide)	102.1 ± 17.1	100.4 ± 15.1	NS	−3.0 ± 4.2	−3.8 ± 3.4	0.56
	Bhandari 2016 [45]	Bariatric surgery	106.89 ± 17.79	117.03 ± 19.89	NR	77.27 ± 10.72	84.89 ± 13.18	NR
	Hutchison 2011 [31]	Intensified exercise training	100.5 ± 4.5 ^!^	96.2 ± 3.5 ^!^	0.42	95.3 ± 4.8 ^!^	96.9 ± 4.5 ^!^	NR
	Kogure 2016 [44]	Progressive resistance training	73.1 ± 15.6	68.1 ± 15.4	NS	0.52 [−0.31, 1.36] ^^^	0.13 [−0.64, 0.90] ^^^	0.14
	Al−Eisa 2017 [46]	Aerobic training	89.8 ± 6.95	84.9 ± 7.2	NR	84.8 ± 6.42	82.2 ± 5.72	NR
**BMI (kg/m^2^)**	Pasquali 2000 [38]	Hypocaloric diet	39.6 ± 6.9	40.1 ± 6.2	NR	38 ± 6.2	37.8 ± 5.7	NR
	Cheang 2016 [43]	Hypocaloric diet	36.6 ± 5.1	35.8 ± 4.8	0.6507	−1.46 [−0.72, −2.20] ^#^	−1.80 [−1.14, −2.45] ^#^	0.4829
	Nikokavoura 2015 [42]	VLCD + behaviour change	40.0 ± 6.3	40.0 ± 6.3	0.955	33.2 ± 6.0	32.8 ± 5.7	NR
	Kowalska 2001 [33]	Hypocaloric diet + Metformin	34.7 ± 6.0	36.2 ± 6.0	NS	31.4 ± 4.8	35.8 ± 7.9	NR
	Pasquali 2000 [38]	Hypocaloric diet + Metformin	39.8 ± 7.9	37.4 ± 3.0	NR	36.4 ± 7.4	32.9 ± 3.4	NR
	Diamanti−Kandarakis 2007 [30]	Hypocaloric diet + AO drug (Orlistat)	35·43 ± 5·31	36·39 ± 6·47	0.58	29.7 ± 4.57	30.15 ± 4.13	NR
	Vosnakis 2013 [36]	Hypocaloric diet + AO drug (Orlistat) + moderate intensity aerobic training	34.83 ± 6.39	36.79 ± 6.98	NR	30.21 ± 5.78	31.01 ± 4.93	NR
	Kahal 2015 [41]	AO drug (Liraglutide)	37.9 ± 5.0	36.5 ± 4.6	NS	−1.0 ± 1.5	−1.4 ± 1.2	0.43
	Villa 1999 [40]	AO drug (Naltrexone)	27.5 ± 6.8	27.4 ± 6.8	NS	26.8 ± 6.7	27 ± 6.8	NR
	Bhandari 2016 [45]	Bariatric surgery	42.52 ± 5.66	45.03 ± 6.3	0.0717	30.76 ± 2.93	32.67 ± 3.51	0.013
	Hutchison 2001 [31]	Intensified exercise training	37.4 ± 1.5 ^!^	35.7 ± 1.3 ^!^	0.43	35 ± 1.6 ^!^	35.9 ± 1.8 ^!^	NR
	Kogure 2016 [44]	Progressive resistance training	28.4 ± 6.0	26.2 ± 5.7	NS	0.21 [−0.11, 0.54] ^	0.05 [−0.25, 0.35] ^	0.08
	Al−Eisa 2017 [46]	Aerobic training	33.45 ± 2.75	31.7 ± 3.8	NR	28.5 ± 2.25	26.8 ± 2.54	NR
**WC (cm)**	Pasquali 2000 [38]	Hypocaloric diet	109 ± 19	109 ± 11	NR	104 ± 13	105 ± 12	NR
	Toscani 2011 [39]	High protein diet	87.74 ± 14.08	83.92 ± 9.13	<0.05	86 ± 12.92	81.83 ± 9.13	NR
	Toscani 2011 [39]	Normal protein diet	93.32 ± 8.05	84.02 ± 9.03	<0.05	90.03 ± 10.41	81.7 ± 11.72	NR
	Kowalska 2001 [33]	Hypocaloric diet + Metformin	98.1 ± 14.8	102.3 ± 13.0	NS	93.4 ± 11.8	100 ± 19.5	NR
	Pasquali 2000 [38]	Hypocaloric diet + Metformin	107 ± 16	102 ± 6	NR	100 ± 15	94 ± 6	NR
	Panidis 2008 [37]	Hypocaloric diet + AO drug (Orlistat) + moderate intensity aerobic training	101.52 ± 2.67	100.53 ± 3.94	NS	87.86 ± 2.29	87.67 ± 2.82	NR
	Hutchison 2011 [31]	Intensified exercise training	106.8 ± 3.4 ^!^	102.8 ± 2.6 ^!^	0.39	103.1 ± 4 ^!^	99.9 ± 4.1 ^!^	NR
	Kogure 2016 [44]	Progressive resistance training	81.7 ± 12.8	76.2 ± 11.3	<0.05	0.86 [0.32, 1.40] ^	0.27 [−0.21, 0.75]	0.21
	Al−Eisa 2017 [46]	Aerobic training	96.2 ± 3.52	94.2 ± 3.82	NR	93.8 ± 3.26	72.7 ± 2.6	NR
**VAT/VF (cm^2^)**	Pasquali 2000 [38]	Hypocaloric diet	121 ± 48	181 ± 94	NR	108 ± 36	159 ± 83	NR
	Pasquali 2000 [38]	Hypocaloric diet + Metformin	151 ± 91	133 ± 38	NR	113 ± 59	100 ± 37	NR
	Hutchison 2011 [31]	Intensified exercise training	129.2 ± 12.8 ^!^	121.5 ± 9.4 ^!^	0.65	107.6 ± 15.1 ^!^	132.7 ± 18.1 ^!^	NR
**SAT/SCFAT (cm^2^)**	Pasquali 2000 [38]	Hypocaloric diet	589 ± 127	554 ± 118	NR	574 ± 111	508 ± 107	NR
	Pasquali 2000 [38]	Hypocaloric diet + Metformin	535 ± 147	554 ± 79	NR	485 ± 170	462 ± 81	NR
	Hutchison 2011 [31]	Intensified exercise training	590.2 ± 35.2 ^!^	550.3 ± 45.2 ^!^	0.49	538.4 ± 40.2 ^!^	558.5 ± 74.5 ^!^	NR

* Between-group difference; ^!^ mean ± SEM; ^ estimated difference [95% confidence interval]; ^#^ mean [95% confidence interval]; AO, anti-obesity; BMI, body mass index; NR, not reported; NS, not significant; PCOS, polycystic ovary syndrome; SAT, subcutaneous adipose tissue area; SCFAT, subcutaneous fat; VAT, visceral adipose tissue area; VF, visceral fat; VLCD, very low calorie diet; WC, waist circumference.

**Table 3:** Reproductive outcomes.

Outcome	References	Intervention	Baseline PCOS: Mean ± SD	Baseline Non-PCOS: Mean ± SD	*p*-Value *	Post-intervention PCOS: Mean ± SD	Post-intervention Non-PCOS: Mean ± SD	*p*-Value *
**Number of ovulations**	Moran 2007 [34]	Hypocaloric diet	NR	NR	NR	1.9 ovulations	1.0 ovulations	<0.001
**Total testosterone (nmol/L)**	Pasquali 2000 [38]	Hypocaloric diet	1.77 ± 0.59	1.32 ± 0.42	<0.05	1.63 ± 0.45	1.14 ± 0.35	NR
	Kowalska 2001 [33]	Hypocaloric diet + Metformin	3.57 ± 1.01	1.91 ± 0.42	<0.05	2.39 ± 1.11	1.7 ± 0.59	NR
	Pasquali 2000 [38]	Hypocaloric diet + Metformin	2.36 ± 1.21	1.46 ± 0.38	<0.01	1.7 ± 0.87	1.25 ± 0.38	NR
	Diamanti-Kandarakis 2007 [30]	Hypocaloric diet + AO drug (Orlistat)	3.01 ± 0.94	1.50 ± 0.43	<0.001	2.28 ± 0.65	1.49 ± 0.36	<0.05
	Panidis 2014 [35]	Hypocaloric diet + AO drug (Orlistat) + moderate intensity aerobic training	2.56 ± 1.00	1.33 ± 0.45	<0.001	2.1 ± 0.78	1.41 ± 0.74	0.006
	Villa 1999 [40]	AO drug (Naltrexone)	1.7 ± 0.5	1.4 ± 0.5	NS	1.9 ± 1.76	1.5 ± 1.2	NR
	Hutchison 2011 [31]	Intensified exercise training	2.9 ± 0.2^!^	1.6 ± 0.2 ^!^	<0.01	2.8 ± 0.3 ^!^	1.8 ± 0.3 ^!^	NR
	Kogure 2016 [44]	Progressive resistance training	3.12 ± 1.22	2.58 ± 1.02	<0.05	0.59 [0.30, 0.89] ^	0.42 [0.16, 0.68] ^	0.15 ^#^
**SHBG (nmol/L)**	Pasquali 2000 [38]	Hypocaloric diet	16.0 ± 7.04	20.2 ± 10.7	NS	13.8 ± 2.1	28.1 ± 14.7	NR
	Kowalska 2001 [33]	Hypocaloric diet + Metformin	32.0 ± 18.3	32.5 ± 16.5	NS	38.6 ± 19.3	36.5 ± 13.3	NR
	Pasquali 2000 [38]	Hypocaloric diet + Metformin	18.7 ± 15.0	23.4 ± 22.7	NS	16.7 ± 8.1	28.9 ± 16.5	NR
	Diamanti-Kandarakis 2007 [30]	Hypocaloric diet + AO drug (Orlistat)	28.72 ± 12.48	40.92 ± 19.54	0.01	37.21 ± 17.59	58.6 ± 27.02	<0.05
	Panidis 2014 [35]	Hypocaloric diet + AO drug (Orlistat) + moderate intensity aerobic training	30.3 ± 13.2	47.8 ± 34.7	0.012	40.3 ± 20.4	62.2 ± 35.5	NS
	Villa 1999 [40]	AO drug (Naltrexone)	30.2 ± 20.4	38.2 ± 16.4	NS	32.5 ± 20.9	39.2 ± 15.7	NR
	Hutchison 2011 [31]	Intensified exercise training	29.0 ± 1.8^!^	43.6 ± 7.8 ^!^	0.04	30.7 ± 2.8 ^!^	54.3 ± 10.6 ^!^	NR
	Kogure 2016 [44]	Progressive resistance training	54.9 ± 37.8	63.0 ± 35.7	NS	0.12 [0.02, 0.23] ^	0.09 [−0.01, 0.18] ^	0.37 ^#^
**FAI**	Kowalska 2001 [33]	Hypocaloric diet + Metformin	14.49 ± 8.49	9.97 ± 7.95	<0.05	9.31 ± 9.95	5.49 ± 3.9	NR
	Panidis 2014 [35]	Hypocaloric diet + AO drug (Orlistat) + moderate intensity aerobic training	10.25 ± 6.31	3_71 ± 2.11	<0.001	6.76 ± 4.32	2.71 ± 1.74	0.021
	Villa 1999 [40]	AO drug (Naltrexone)	9.3 ± 6.5	4.9 ± 3.1	<0.05	8.9 ± 5.7	5.2 ± 3.3	NR
	Hutchison 2011 [31]	Intensified exercise training	10.7 ± 1.1 ^!^	4.6 ± 0.9 ^!^	<0.01	10.1 ± 1.6 ^!^	4.1 ± 1.1 ^!^	NR
	Kogure 2016 [44]	Progressive resistance training	8.3 ± 6.3	5.6 ± 4.6	<0.05	0.98 [−0.03, 1.99] ^	0.37 [−0.50, 1.24] ^	0.25 ^#^

* Between-group difference; ^!^ mean ± SEM; ^ estimated difference [95% confidence interval]; ^#^ *p* value adjusted for age, BMI, and HOMA-IR; AO, anti-obesity; FAI, free androgen index; NR, not reported; NS, not significant; PCOS, polycystic ovary syndrome; SHBG, sex hormone-binding globulin.

**Table 4:** Glucose and insulin homeostasis.

Outcome	References	Intervention	Baseline PCOS: Mean ± SD	Baseline Non-PCOS: Mean ± SD	*p*-Value *	Post-Intervention PCOS: Mean ± SD	Post-Intervention Non-PCOS: Mean ± SD	*p*-Value *
**Fasting glucose (mmol/L)**	Pasquali 2000 [38]	Hypocaloric diet	5.61 ± 1.0	5.11 ± 0.56	NS	5.27 ± 0.61	5.16 ± 0.94	NR
	Cheang 2016 [43]	Hypocaloric diet	4.75 ± 0.45	4.67 ± 0.21	0.60	−0.06 (−0.25, 0.12) ^%^	0.01 (−0.13, 0.15) ^%^	0.5041
	Toscani 2011 [39]	High protein diet	4.97 ± 0.34	4.95 ± 0.45	NS	5.02 ± 0.40	5.05 ± 0.54	NR
	Toscani 2011 [39]	Normal protein diet	4.90 ± 0.36	4.98 ± 0.41	NS	4.98 ± 0.35	5.02 ± 0.35	NR
	Pasquali 2000 [38]	Hypocaloric diet + Metformin	5.49 ± 1.61	4.94 ± 0.56	NS	5 ± 0.94	4.94 ± 0.72	NR
	Diamanti-Kandarakis 2007 [30]	Hypocaloric diet + AO drug (Orlistat)	5.72 ± 0.51	5.77 ± 0.61	0.92	5.52 ± 0.47	5.69 ± 0.52	NR
	Panidis 2014 [35]	Hypocaloric diet + AO drug (Orlistat) + moderate intensity aerobic training	5.58 ± 0.57	5.81 ± 0.66	NS	5.28 ± 0.56	5.58 ± 0.51	NS
	Villa 1999 [40]	AO drug (Naltrexone)	4.65 ± 0.33	4.55 ± 0.22	NS	4.33 ± 0.56	4.48 ± 0.37	NR
	Hutchison 2011 [31]	Intensified exercise training	5.0 ± 0.1 ^!^	4.8 ± 0.1 ^!^	0.57	4.9 ± 0.1 ^!^	4.9 ± 0.1 ^!^	NR
	Kogure 2016 [44]	Progressive resistance training	5.34 ± 0.91	5.31 ± 0.97	NS	0.37 [0.08, 0.66] $	0.30 [0.04, 0.56] $	0.12 **
	Al-Eisa 2017 [46]	Aerobic training	4.50 ± 2.80	5.59 ± 1.57	NR	4.50 ± 2.80	5.59 ± 1.57	NR
**OGTT-glucose (mmol/L)**	Toscani 2011 [39]	High protein diet	6.27 ± 1.60	5.02 ± 0.99	NR	6.92 ± 2.0	5.63 ± 1.56	NR
	Toscani 2011 [39]	Normal protein diet	6.55 ± 1.50	5.41 ± 1.02	NR	6.64 ± 2.16	5.18 ± 1.02	NR
	Diamanti-Kandarakis 2007 [30]	Hypocaloric diet + AO drug (Orlistat)	6.35 ± 1.35	6.54 ± 1.54	0.65	6.17 ± 0.85	6.21 ± 1.77	NR
**Fasting insulin (pmol/L)**	Pasquali 2000 [38]	Hypocaloric diet	240.36 ± 214.53	149.24 ± 79.64	NS	136.33 ± 103.32	103.32 ± 76.06	NR
	Toscani 2011 [39]	High protein diet	76.05 (60.00–123.41) ^	57.85 (28.20–84.94) ^	<0.05	61.67 (52.71–108.83) ^	56.74 (33.20–97.92) ^	NR
	Toscani 2011 [39]	Normal protein diet	128.83 (93.06–213.28) ^	59.59 (41.04–84.52) ^	<0.05	129.15 (76.99–233.04) ^	48.00 (31.14–80.14) ^	NR
	Pasquali 2000 [38]	Hypocaloric diet + Metformin	308.53 ± 218.12	217.40 ± 58.84	NS	154.98 ± 223.86	102.6 ± 60.99	NR
	Kowalska 2001 [33]	Hypocaloric diet + Metformin	26.2 ± 13.9 ^#^	18.3 ± 14.2 ^#^	<0.05	16.9 ± 9.1 ^#^	20.0 ± 13.5 ^#^	NR
	Diamanti-Kandarakis 2007 [30]	Hypocaloric diet + AO drug (Orlistat)	127.37 ± 61.12	125.5 ± 87.09	0.45	76.4 ± 34.93	77.02 ± 47.3	NR
	Panidis 2014 [35]	Hypocaloric diet + AO drug (Orlistat) + moderate intensity aerobic training	129.87 ± 75.01	133.34 ± 161.82	NS	81.08 ± 50.94	68.16 ± 40.18	NS
	Villa 1999 [40]	AO drug (Naltrexone)	99.7 ± 75	104.3 ± 63.1	NS	90.4 ± 61	85.3 ± 39.4	NR
	Hutchison 2011 [31]	Intensified exercise training	141.6 (100.8–181.2) ^	72.6 (58.8–115.8) ^	0.02	97.8 (66.6–231.0) ^	115.2 (76.2–177.6) ^	NR
	Kogure 2016 [44]	Progressive resistance training	64.59 ± 47.92	36.11 ± 31.25	<0.05	0.90 [−0.21, 1.94] ^$^	−0.90 [−1.88, 0.07] ^$^	0.58 **
	Al-Eisa 2017 [46]	Aerobic training	17.8 ± 4.20 ^@^	20.6 ± 8.2 ^@^	NR	14.8 ± 2.9 ^@^	16.1 ± 5.1 ^@^	NR

* Between-group difference; ^!^ mean ± SEM; ^ median (IQ range); ^#^ IU/L; ^@^ data reported as mU/mL (as per original study); ^%^ mean (95% confidence interval); ^$^ estimated difference [95% confidence interval]; ** *p* value adjusted for age, BMI, and HOMA-IR; AO, anti-obesity; f-glucose, fasting glucose; f-insulin, fasting insulin; NR, not reported; NS, not significant; OGTT-glucose, oral glucose tolerance test glucose; PCOS, polycystic ovary syndrome.

**Table 5:** Lipids and blood pressure.

Outcome	References	Intervention	Baseline PCOS: Mean ± SD	Baseline Non-PCOS: Mean ± SD	*p*-Value *	Post-intervention PCOS: Mean ± SD	Post-intervention Non-PCOS: Mean ± SD	*p*-Value *
**Total cholesterol (mmol/L)**	Toscani 2011 [39]	High protein diet	4.60 ± 1.15	4.24 ± 0.71	NR	4.32 ± 1.12	4.19 ± 0.82	NR
	Toscani 2011 [39]	Normal protein diet	4.31 ± 1.04	4.05 ± 1.13	NR	4.02 ± 0.82	3.86 ± 1.36	NR
	Panidis 2014 [35]	Hypocaloric diet + AO drug (Orlistat) + moderate intensity aerobic training	5.04 ± 1.00	5.48 ± 0.93	NS	4.52 ± 0.82	4.55 ± 0.87	<0.001
	Hutchison 2011 [31]	Intensified exercise training	5.0 ± 0.3 ^!^	4.7 ± 0.2 ^!^	0.81	4.4 ± 0.2 ^!^	4.8 ± 0.4 ^!^	NR
**LDL cholesterol (mmol/L)**	Toscani 2011 [39]	High protein diet	3.91 ± 1.09	3.66 ± 0.61	NR	3.78 ± 1.1	3.54 ± 0.76	NR
	Toscani 2011 [39]	Normal protein diet	3.62 ± 0.95	3.30 ± 1.11	NR	3.33 ± 0.75	3.13 ± 1.34	NR
	Panidis 2014 [35]	Hypocaloric diet + AO drug (Orlistat) + moderate intensity aerobic training	3.30 ± 0.90	3.65 ± 0.68	NS	2.87 ± 0.84	2.98 ± 0.47	0.001
	Hutchison 2011 [31]	Intensified exercise training	3.3 ± 0.2 ^!^	3.0 ± 0.2 ^!^	0.48	3 ± 0.2 ^!^	3.1 ± 0.4 ^!^	NR
**HDL cholesterol (mmol/L)**	Toscani 2011 [39]	High protein diet	1.30 ± 0.19	1.35 ± 0.38	NR	1.27 ± 0.2	1.34 ± 0.39	NR
	Toscani 2011 [39]	Normal protein diet	1.19 ± 0.32	1.39 ± 0.26	NR	1.18 ± 0.32	1.5 ± 0.25	NR
	Panidis 2014 [35]	Hypocaloric diet + AO drug (Orlistat) + moderate intensity aerobic exercise	1.20 ± 0.22	1.32 ± 0.31	NS	1.21 ± 0.23	1.21 ± 0.26	0.006
	Hutchison 2011 [31]	Intensified exercise training	1.0 ± 0.1 ^!^	1.2 ± 0.1 ^!^	0.04	1 ± 0.1 ^!^	1.2 ± 0.1 ^!^	NR
**Triglycerides (mmol/L)**	Toscani 2011 [39]	High protein diet	0.86 (0.47–1.47) ^	0.68 (0.47–0.76) ^	NR	0.55 (0.49–0.71) ^	0.92 (0.55–1.02) ^	NR
	Toscani 2011 [39]	Normal protein diet	0.97 (0.67–1.30) ^	0.71 (0.41–1.62) ^	NR	0.94 (0.81–1.15) ^	0.88 (0.64–1.21) ^	NR
	Panidis 2014 [35]	Hypocaloric diet + AO drug (Orlistat) + moderate intensity aerobic training	1.17 ± 0.56	1.28 ± 0.62	NS	0.99 ± 0.44	0.87 ± 0.25	NS
	Hutchison 2011 [31]	Intensified exercise training	1.4 ± 0.2 ^!^	1.2 ± 0.2 ^!^	0.46	0.9 ± 0.1 ^!^	1.3 ± 0.1 ^!^	NR
**BP systolic (mmHg)**	Toscani 2011 [39]	High protein diet	125.7 ± 19.0	116.1 ± 10.41	NR	126 ± 23.1	117.85 ± 10.18	NR
	Toscani 2011 [39]	Normal protein diet	119.1 ± 16.4	116.43 ± 10.3	NR	119.36 ± 15.38	110.71 ± 7.32	NR
	Harrison 2012 [32]	Intensified exercise training	108 ± 14.6 ^!^	118 ± 16.7 ^!^	NS	109 ± 10.4 ^!^	116 ± 16.2 ^!^	NR
**BP diastolic (mmHg)**	Toscani 2011 [39]	High protein diet	77.9 ± 10.75	74.6 ± 8.46	NR	80 ± 11.2	74 ± 8.83	NR
	Toscani 2011 [39]	Normal protein diet	78 ± 11.83	75.14 ± 9.6	NR	77.82 ± 12.02	72.57 ± 7.72	NR
	Harrison 2012 [32]	Intensified exercise training	72 ± 10.2 ^!^	75 ± 8.8 ^!^	NS	69 ± 7.4 ^!^	73 ± 10.5 ^!^	NR

* Between-group difference; ^!^ mean ± SEM; ^ median (IQ range); AO, anti-obesity; BP, blood pressure; HDL, high-density lipoprotein; LDL, low-density lipoprotein; NR, not reported; NS, not significant; PCOS, polycystic ovary syndrome.

## Supplementary Materials

The following are available online at www.mdpi.com/2072-6643/9/9/996/s1, Table S1: Selection criteria, Table S2: Search terms, Table S3: Excluded studies, Table S4: Critical appraisals of included studies.
