# Supplementary material for: Weight Management Interventions in Women with and without PCOS: A Systematic Review
Source: Nutrients. 2017 Sep 8;9(9):996. doi: 10.3390/nu9090996 (PMC5622756; doi:10.3390/nu9090996)
Supplement: Supplementary file 1 [file nutrients-09-00996-s001.zip › nutrients-214864-supplementary.pdf]

Supplementary table S1. Selection criteria

	Include	Exclude
Population	Women with PCOS according to any diagnosis or self-report<br>Any age<br>Any weight<br>Any medication or existing diseases providing these are documented appropriately.	Condition with reproductive symptoms similar to PCOS, including congenital adrenal hyperplasia, Cushings syndrome, hyperprolactinemia, thyroid disease, and androgen secreting tumours
Comparative group	Women without PCOS (clinically diagnosed or self-report)<br>Any age<br>Any weight<br>Any medication or existing diseases providing these are documented appropriately.	Condition with reproductive symptoms similar to PCOS, including congenital adrenal hyperplasia, Cushings syndrome, hyperprolactinemia, thyroid disease, and androgen secreting tumours
Intervention	Weight management interventions including all types of lifestyle interventions (all types of dietary compositions, all types of exercise, behavioural interventions), anti-obesity medication (bupropion/naltrexone, orlistat, liraglutide, lorcaserin, phentermine/topiramate), metformin, acupuncture or bariatric surgery.<br>All study durations.	
Outcomes	Primary<br>*Anthropometric outcomes:* weight<br><br>Secondary<br>*Anthropometric outcomes:* BMI, Fat distribution (WC, HC or WHR), Fat and lean mass (central/truncal and total) measured using BIA, DEXA, MRI, or CT.<br>*Fertility outcomes*: Pregnancy, Live birth, Miscarriage, Menstrual regularity/ovulation	

	Reproductive nonfertility outcomes: Reproductive hormonal parameters (Total testosterone, SHBG, Free testosterone) and Clinical Hyperandrogenism (hirsutism assessed clinically by Ferriman-Gallwey score)<br>*Metabolic outcomes:* Insulin resistance (Fasting insulin, HOMA or other measures e.g. OGTT-insulin, Euglycemic hyperinsulinemic clamp, GIR, GDR, FSIVGTT, Insulin sensitivity), Fasting glucose, OGTT- glucose, HbA1c, Lipid profile (TC, HDL-C, LDL-C, TG), BP, hs-CRP<br>*Coagulation:* Fibrinogen and PAI<br>*Inflammatory markers*: IL-6, TNF-α<br>*Changes in health related quality of life*<br>*Changes in symptoms of anxiety and depression*	
Study type	All study types	
Language	English	
Publication date	Any	

BIA, bioelectrical impedance analysis; BP, blood pressure; BMI, body mass index; CT, computerized tomography; DEXA, dual x-ray absorptiometry; FSIVGTT, standard frequently-sampled intravenous glucose tolerance test; GDR, glucose disposal rate; GIR, glucose infusion rate; HDL-C, high-density lipoprotein cholesterol; HC, hip circumference; HOMA, homeostatic model assessment; hs-CRP, high sensitive C-reactive protein; IL-6, interleukin-6; LDL-C, low-density lipoprotein cholesterol; MRI, magnetic resonance imaging; OGTT, oral glucose tolerance test; PAI, plasminogen activator inhibitor; PCOS, polycystic ovary syndrome; SHBG, sexual-hormone binding globulin; TC, total cholesterol; TG, triglycerides; TNF-α, tumor necrosis factor –α; WC, waist circumference; WHR, waist-hip-rati

Supplementary table S3: excluded studies

Citation	Reason for exclusion
Anonymous (2001). "Metformin useful for obese anovulatory women with PCOS." Contemporary OB/GYN 46(4): 148-148.	Not comparing PCOS with non-PCOS.
Bates, G. W. and N. S. Whitworth (1982). "Effect of body weight reduction on plasma androgens in obese, infertile women." Fertility & Sterility 38(4): 406-409.	Not comparing PCOS with non-PCOS.
Chiofalo, F., et al. (2017). "Bariatric Surgery Reduces Serum Anti-mullerian Hormone Levels in Obese Women With and Without Polycystic Ovarian Syndrome." Obesity Surgery: 1-5.	Mean and standard deviations not presented after surgery for outcomes of interest.
Cinar, N., et al. (2013). "Ethinyl estradiol-drospirenone vs ethinyl estradiol-drospirenone plus metformin in the treatment of lean women with polycystic ovary syndrome." Clinical endocrinology 78(3): 379-384.	Not comparing PCOS with non-PCOS.
Cortet-Rudelli, C. and D. Dewailly (1998). "How actual is the dietary treatment in overweighting patients with polycystic ovary syndrome?" Journal of endocrinological investigation 21(9): 636-640.	No original data (e.g. letter, editorial, non-systematic review).
Gamus, D. (2011). "Electro-acupuncture in polycystic ovary syndrome: A potent placebo or a new promising treatment?" Focus on Alternative and Complementary Therapies 16(3): 229-230.	Not comparing PCOS with non-PCOS.
Georgopoulos, N. A., et al. (2009). "Effect of sibutramine on weight reduction and insulin resistance in women with polycystic ovary syndrome." Fertility & Sterility 91(6): e1.	No original data (e.g. letter, editorial, non-systematic review).

Supplementary table S2: Search terms

1	exp polycystic ovary syndrome/
2	polycystic ovar$.mp.
3	poly-cystic ovar$.mp.
4	PCO$.mp.
5	(stein-leventhal or leventhal).mp.
6	anovulation/
7	anovulat$.mp.
8	oligo-ovulat$.mp.
9	oligoovulat$.mp.
10	(ovar$ adj5 (sclerocystic or polycystic or poly-cystic or degenerat$ or hyperandrogen$ or hyper-androgen$)).mp.
11	or/1-10
12	diet$.mp.
13	nutrition$.mp.
14	meal$.mp.
15	food$.mp.
16	(Energy adj3 restrict$).mp.
17	(Energy adj3 reduc$).mp.
18	kilojoule$.mp.
19	calor$.mp.
20	hypocaloric.mp.
21	Feeding behaviour$.mp.
22	Feeding behavior$.mp.
23	eating behaviour$.mp.

Glintborg, D., et al. (2015). "Increased	Not comparing PCOS with non-PCOS.

24 eating behavior$.mp.

25 exp diet/

26 exp diet therapy/

27 exp nutrition therapy/

28 exp food/

29 exp feeding behavior/

30 (diet$ or diet$ therap$ or diet$ modification$ or diet$ intervention$ or diet$ counsel$).mp.

31 exp Food Habits/

32 isocaloric.tw.

33 Energy Intake/

34 or/12-33

35 exercise$.mp.

36 exercise therapy.mp.

37 exertion.mp.

38 physical fitness.mp.

39 physical activit$.mp.

40 physical performance.mp.

41 sport$.mp.

42 (strength adj2 training).mp.

43 resistance training.mp.

44 (aerobic$ adj2 training).mp.

45 (endurance adj training).mp.

46 physical training.mp.

thrombin generation in women with	

47 (strength$ adj2 exercise$).mp.

48 (weight-bearing adj2 exercise$).mp.

49 (Resistance adj2 exercise$).mp.

50 (Aerobic$ adj2 exercise$).mp.

51 (Endurance adj2 exercise$).mp.

52 (Physical adj2 exercise$).mp.

53 exp exercise/

54 exp exercise therapy/

55 physical exertion/

56 exp sports/

57 exp physical endurance/

58 exp Yoga/

59 exp sports/ or exp bicycling/ or exp running/ or exp swimming/ or exp walking/

60 exp Physical Fitness/

61 or/35-60

62 exp cognitive therapy/

63 exp Psychophysiology/

64 exp relaxation techniques/

65 exp relaxation technique/

66 exp Relaxation Therapy/

67 (cognitive adj2 therap$).mp.

68 (relax$ adj2 technique$).mp.

69 relax$.mp.

70 exp Meditation/

71 kinesiotherap$.mp.

72 exp Psychotherapy/

73 Psychotherap$.mp.

74 exp Behavior Therapy/

75 (Behavio$ adj2 therap$).mp.

76 risk reduction behavior/

77 (risk reduction adj2 behavio$).mp.

78 behavior control/

79 (behavio$ adj2 control).mp.

80 exp Behavior/

81 behavio?r.mp.

82 exp health behavior/

83 (health adj2 behavio$).mp.

84 behavio?r$ coping strateg$.mp.

85 or/62-84

86 exp life style/

87 exp life change events/

88 (life*style adj2 change$).mp.

89 (life*style adj2 intervention$).mp.

90 (life*style adj2 modif$).mp.

91 (life*style adj2 choice$).mp.

92 (life?style or life style).mp.

93 life?style program.mp.

94 wellness.mp.

on the effect of metformin and oral	

95 (well being or well?being).mp.

96 or/86-95

97 exp Health Promotion/

98 (Health adj2 Promotion).mp.

99 exp Health Education/

100 (Health$ adj2 Education).mp.

101 patient education as topic/ or prenatal education/

102 (motivation$ adj2 therap$).mp.

103 educat$.mp.

104 advice.mp.

105 counseling/ or directive counseling/

106 counsel$.mp.

107 inform$.mp.

108 promotion$.mp.

109 campaign$.mp.

110 health.mp.

111 (healthcar$ or health-car$ or health car$).mp.

112 or/97-111

113 Metformin/

114 metformin.tw.

115 glucophage.tw.

116 dimethylbiguanidium.tw.

117 dimethylguanylguanidine.tw.

118 or/113-117

contraceptives." Metabolism: Clinical &	

119 exp Bariatric Surgery/

120 bariatric surgery.tw.

121 exp Laparoscopy/ or exp Gastroplasty/ or exp Gastric Bypass/

122 exp Surgical Stapling/

123 exp Gastrectomy/ or exp Biliary Tract Surgical Procedures/

124 exp Biliopancreatic Diversion/

125 exp Gastric Balloon/

126 laparoscop$ adjust$ gastric band$.tw.

127 LAGB.tw.

128 LASGB.tw.

129 laparoscop$ band$.tw.

130 lap-band$.tw.

131 lap band$.tw.

132 gastric band$.tw.

133 swedish band$.tw.

134 SAGB.tw.

135 gastroplast$.tw.

136 VBG.tw.

137 gastric stapl$.tw.

138 roux-en-Y.tw.

139 RYGP.tw.

140 gastric bypass.tw.

141 biliopancreatic diversion.tw.

142 BPD.tw.

Experimental **64**(10): 1272-1278.	

143 scopinaro.tw.

144 gastrectomy.tw.

145 gastric plication.tw.

146 duodenal switch.tw.

147 DS.tw.

148 BPD-DS.tw.

149 gastric balloon.tw.

150 or/119-149

151 exp Acupuncture/

152 exp acupuncture therapy/ or exp acupressure/ or exp acupuncture analgesia/ or exp acupuncture, ear/ or exp electroacupuncture/ or exp meridians/ or exp moxibustion/

153 acupressure$.tw.

154 Acupuncture.tw.

155 (electroacupuncture or electro-acupuncture).tw.

156 meridian$.tw.

157 mox$.tw.

158 (shiatsu or tui na).tw.

159 needling.tw.

160 shu.tw.

161 acup$ point$.tw.

162 or/151-161

163 exp Anti-Obesity Agents/

164 anti-obesity agent$.tw.

165 anti-obesity drug$.tw.

Goss, A. M. (2014). "Beneficial effects of a	This is a report about a study and not the

166 exp Bupropion/

167 bupropion$.tw.

168 exp Naltrexone/

169 naltrexone.tw.

170 contrave.tw.

171 orlistat.tw.

172 xenical.tw.

173 alli.tw.

174 liraglutide.tw.

175 saxenda.tw.

176 victoza.tw.

177 lorcaserin.tw.

178 belviq.tw.

179 phentermin.tw.

180 topiramate.tw.

181 qsymia.tw.

182 or/163-181

183 34 or 61 or 85 or 96 or 112 or 118 or 150 or 162 or 182

184 body weight changes/ or weight gain/ or weight loss/

185 ((weight or BMI or body mass index) and (prevent$ or preser$ or maintain$ or management or maintenance or reduc$ or los$ or decreas$ or control)).tw

186 or/184-185

187 11 and 183 and 186

188 limit 187 to (english language and humans)

reduced carbohydrate diet in PCOS."	actual study. The study is not comparing

Nutrition Close-Up: 4-6.	PCOS with non-PCOS.
Gower, B. A., and Goss, A. M. (2015). “A lower-carbohydrate, higher-fat diet reduces abdominal and intermuscular fat and increases insulin sensitivity in adults at risk of type 2 diabetes.” Journal of Nutrition **145**(1): 177S-183S.	Men were included in comparison group, results not presented by sex. Also interventions differed slightly for PCOS and non-PCOS groups.
Guido, M., et al. (2006). "Role of opioid antagonists in the treatment of women with glucoregulation abnormalities." Current Pharmaceutical Design **12**(8): 1001-1012.	No original data (e.g. letter, editorial, non-systematic review).
Hulchiy, M., Nybacka, A., Sahlin, L., & Hirschberg, A. L. (2016). Endometrial Expression of Estrogen Receptors and the Androgen Receptor in Women With Polycystic Ovary Syndrome: A Lifestyle Intervention Study. Journal of Clinical Endocrinology & Metabolism **101**(2): 561-571.	Only women in the PCOS group undertook the intervention
Joham, A. E., et al. (2012). "Pigment epithelium-derived factor, insulin sensitivity, and adiposity in polycystic ovary syndrome: impact of exercise training." Obesity **20**(12): 2390-2396.	Substudy of the included study Hutchison 2011. This article did not provide any additions to the outcomes and therefore it was excluded from the systematic review.
Khademi, A., et al. (2010). "The Effect of Exercise in PCOS Women Who Exercise Regularly." Asian Journal of Sports Medicine **1**(1): 35-40.	This study compared history of exercise in women with and without PCOS. No controlled intervention was executed and therefore the study was excluded.
Kiddy, D. S., et al. (1989). "Diet-induced changes in sex hormone binding globulin and free testosterone in women with normal or polycystic ovaries: correlation with serum insulin and insulin-like growth factor-I." Clinical endocrinology **31**(6): 757-763.	Compared women with and without Polycystic ovaries (PCO) and not with and without PCOS.
Kucur, S. K., et al. (2015). How medical treatment affects mean platelet volume as a cardiovascular risk marker in polycystic ovary syndrome? Blood Coagulation and Fibrinolysis **26**(8): 862-865.	Only women in the PCOS group undertook the intervention
Lim, S. S., et al. (2007). "Obesity	No original data (e.g. letter, editorial,

management in women with polycystic ovary syndrome." Women's health 3(1): 73-86.	non-systematic review).
Moini, A., et al. (2015). "Effect of orlistat on weight loss, hormonal and metabolic profiles in women with polycystic ovarian syndrome: a randomized double-blind placebo-controlled trial." Endocrine 49(1): 286-289.	No comparing women with and without PCOS.
Moran, L. and R. J. Norman (2004). "Understanding and managing disturbances in insulin metabolism and body weight in women with polycystic ovary syndrome." Best Practice & Research in Clinical Obstetrics & Gynaecology 18(5): 719-736.	No original data (e.g. letter, editorial, non-systematic review).
Moran, L. J., et al. (2006). "Effects of lifestyle modification in polycystic ovarian syndrome." Reproductive Biomedicine Online 12(5): 569-578.	No original data (e.g. letter, editorial, non-systematic review).
Moran, L. J., et al. (2011). "Exercise decreases anti-mullerian hormone in anovulatory overweight women with polycystic ovary syndrome: a pilot study." Hormone & Metabolic Research 43(13): 977-979.	Substudy of the included study Hutchison et al. 2011. This article did not provide any additional outcomes of interest for this review and was therefore excluded.
Moran, L. J., et al. (2010). "Polycystic ovary syndrome and weight management." Women's health 6(2): 271-283.	No original data (e.g. letter, editorial, non-systematic review).
Moran, L. J., et al. (2010). "The effect of modifying dietary protein and carbohydrate in weight loss on arterial compliance and postprandial lipidemia in overweight women with polycystic ovary syndrome." Fertility and sterility 94(6): 2451-2454.	Not comparing women with and without PCOS.
Moran, L. J., et al. (2013). "The contribution of diet, physical activity and sedentary behaviour to body mass index in women with and without polycystic ovary syndrome." Human Reproduction 28(8): 2276-2283.	Population based observational study with data collected at 13 year follow up. No controlled intervention was executed and therefore this study was excluded from this review.

Oktenli, C., et al. (2007). "Metformin decreases circulating acylation-stimulating protein levels in polycystic ovary syndrome." Gynecological endocrinology **23**(12): 710-715.	Not comparing women with and without PCOS.
Pasquali, R. and A. Gambineri (2006). "Insulin-sensitizing agents in polycystic ovary syndrome." European Journal of Endocrinology **154**(6): 763-775.	No original data (e.g. letter, editorial, non-systematic review).
Pau, C. T., et al. (2016). "The role of variants regulating metformin transport and action in women with polycystic ovary syndrome." Pharmacogenomics **17**(16): 1765-1773.	Did not have a comparison group of women without PCOS
Paulson, M., Sahlin, L., and Hirschberg, A. L. (2016). "Progesterone receptors and proliferation of the endometrium in obese women with polycystic ovary syndrome - a lifestyle intervention study." Journal of Clinical Endocrinology & Metabolism **102**(4): 1244-1253.	Intervention only in the PCOS group
Raisbeck, E. (2008). "Ensuring treatment for polycystic ovaries." Practice Nursing **19**(8): 395-398.	No original data (e.g. letter, editorial, non-systematic review).
Raja-Khan, N., et al. (2015). "Mindfulness-based stress reduction for overweight/obese women with and without polycystic ovary syndrome: design and methods of a pilot randomized controlled trial." Contemporary clinical trials **41**: 287-297.	Pilot study. No results are provided in this report.
Sahin, I., et al. (2004). "Metformin versus flutamide in the treatment of metabolic consequences of non-obese young women with polycystic ovary syndrome: a randomized prospective study." Gynecological endocrinology **19**(3): 115-124.	Not comparing women with and without PCOS.
Sanders, M. E. (2012). "On the Floor. Tailored Exercise for Polycystic Ovary Syndrome Participants." ACSM's Health & Fitness Journal **16**(4): 29-32.	No original data (e.g. letter, editorial, non-systematic review).
Scott, D., et al. (2016). "Associations of	Post intervention results grouped by

Vitamin D with Inter- and Intra-Muscular Adipose Tissue and Insulin Resistance in Women with and without Polycystic Ovary Syndrome." Nutrients **8**(12): 774.	Vitamin D status, rather than PCOS vs non-PCOS status
Singh, S., et al. (2012). "Plasma adiponectin levels in women with polycystic ovary syndrome: impact of metformin treatment in a case-control study." Diabetes & Metabolic Syndrome **6**(4): 207-211.	Not comparing women with and without PCOS.
Smith, J. W. and J. S. Taylor (2011). "Polycystic ovary syndrome: evidence-based strategies for managing symptoms and preventing long-term sequelae." Nursing for Women's Health **15**(5): 402-410; quiz 411.	No original data (e.g. letter, editorial, non-systematic review).
Sun, X., Wu, X., Zhou, Y., Yu, X., and Zhang, W. (2015). "Evaluation of Apelin and Insulin Resistance in Patients with PCOS and Therapeutic Effect of Drospirenone-Ethinylestradiol Plus Metformin." Medical Science Monitor **21**: 2547-2552.	Intervention only in the PCOS group
Tarkun, I., et al. (2010). "Impact of treatment with metformin on adipokines in patients with polycystic ovary syndrome." European cytokine network **21**(4): 272-277.	Not comparing women with and without PCOS.
Teede, H., et al. (2010). "Polycystic ovary syndrome: a complex condition with psychological, reproductive and metabolic manifestations that impacts on health across the lifespan." BMC Medicine **8**: 41.	No original data (e.g. letter, editorial, non-systematic review).
Turner-McGrievy, G., Davidson, C. R., and Billings, D. L. (2015). "Dietary intake, eating behaviors, and quality of life in women with polycystic ovary syndrome who are trying to conceive." Human Fertility **18**(1): 16-21.	Comparison group data is from other studies, and are baseline data
Victor, V. M., et al. (2015). « Effects of metformin on mitochondrial function of leukocytes from polycystic ovary	Only the PCOS group underwent the intervention

syndrome patients with insulin resistance." European Journal of Endocrinology **173**(5): 683-691.	
Victor, V. M., et al. (2015). "Metformin modulates human leukocyte/endothelial cell interactions and proinflammatory cytokines in polycystic ovary syndrome patients." Atherosclerosis **242**(1): 167-173.	Metformin regime not reported for comparison T2DM group
Yilmaz, M., et al. (2005). "The effects of rosiglitazone and metformin on oxidative stress and homocysteine levels in lean patients with polycystic ovary syndrome." Human reproduction (Oxford, England) **20**(12): 3333-3340.	Not comparing women with and without PCOS.

Supplementary table S4: critical appraisals of included studies

**Study ID**	Diamanti-Kandarakis 2007
**Study citation**	Diamanti-Kandarakis, E., et al. (2007). "Effect of long-term orlistat treatment on serum levels of advanced glycation end-products in women with polycystic ovary syndrome." Clinical endocrinology **66**(1): 103-109.
**EXTERNAL VALIDITY – IS THIS STUDY AND ITS RESULTS GENERALIZABLE TO MY SYSTEMATIC REVIEW QUESTION?**	
**Patient/population/ participants**	Women with PCOS, age 27.52±5.77 years and BMI 35.43±5.31 kg/m^2.
**Control population**	Women without PCOS, age 32.06±5.64 years and BMI 36.39±6.47 kg/m^2.
**N**	The number of participants that were:<br>• Screened: 65 (40 PCOS 25 control)-discussed treatment and side effects.<br>• Enrolled: 47 (29 PCOS 18 control)-agreed to participate<br>• Assessed: 47 (29 PCOS 18 control)<br>• Followed up: -
**Setting**	"The study was conducted in collaboration with the Ippokrateion Hospital of Thessaloniki, the Laiko University Hospital and the Laboratory of Biological Chemistry of the University of Athens Medical School."

**Intervention/indicator**	"At the initial evaluation (baseline), the basic metabolic rate (BMR in kcal/day) of all women was calculated and adjusted for moderate daily physical activity as follows:<br><br>18-30 years old: [0.0621 x weight (kg) + 2·0357] x 240 x 1.3<br>> 31 years old: [0.0342 x weight (kg) + 3·5377] x 240 x 1·3<br><br>All subjects were prescribed a normal-protein, energy-restricted diet [BMR 600 kcal/day, 50% as carbohydrate, 30% as fat (10% saturated), 20% as protein], for a period of 24 weeks. In addition, orlistat (Xenical®, Roche) was administered at a dose of 120 mg three times daily, before each meal, for the same period."	
**Outcomes**	• BMI<br>• WHR<br>• Testosterone<br>• SHBG<br>• AGE plasma levels (not relevant to systematic review)<br>• Serum fasting insulin<br>• GLU 0 min (not relevant to systematic review)<br>• 120 minute glucose<br>• Fasting glucose to insulin ratio<br>• HOMA-IR<br>• QUICKI (not relevant to systematic review)	
**Does the study have a clearly focused question and/or PICO?**	Yes	
**Inclusion Criteria**	Yes	"All of the participants were in good health and for at least 3 months before the study were off medication known to affect carbohydrate or sex hormone metabolism, including oral contraceptive agents.<br>PCOS diagnosis was based on the Rotterdam criteria, that is on the presence of two of the following three criteria: (i) oligo- and/or anovulation (< 8 menses per year); (ii) clinical and/or biochemical signs of hyperandrogenism; (iii) polycystic ovaries on ultrasonography, and exclusion of related disorders (nonclassical congenital adrenal hyperplasia, androgensecreting neoplasms, thyroid disease and hyperprolactinaemia).

<table>
<tr><td colspan="2"></td><td></td><td>The women in the control group had normal ovulating cycles (28±2 days, blood progesterone levels > 10 ng/ml in two consecutive cycles), no hyperandrogenaemia, or signs of hyperandrogenism (hirsutism, acne or alopecia) on physical examination, normal sonographic appearance of the ovaries (controls) and had not sought treatment for menstrual disturbances or infertility at any time.”</td></tr>
<tr><td colspan="2">Exclusion Criteria</td><td>Yes</td><td>“None of the women studied had galactorrhoea, nor any systemic disease that could affect their reproductive physiology.”</td></tr>
<tr><td colspan="2">If there were specified inclusion/ exclusion criteria, were these appropriate?</td><td>Yes</td><td></td></tr>
<tr><td colspan="2">Is a case control study the appropriate design to answer this question?</td><td>Yes</td><td></td></tr>
<tr><td colspan="2">Were the outcomes measured appropriate?</td><td>Yes</td><td></td></tr>
<tr><td colspan="2">Was there sufficient duration of follow-up for outcomes to occur?</td><td>Yes</td><td></td></tr>
<tr><td colspan="4">INTERNAL VALIDITY – HAS THIS STUDY BEEN CONDUCTED RIGOROUSLY IN ORDER TO REDUCE BIAS?</td></tr>
<tr><td rowspan="2">SELECTION BIAS</td><td>Were the cases and controls taken from comparable populations?</td><td>Not reported</td><td></td></tr>
<tr><td>Was the case definition adequate and established in a standard, valid and reliable way?</td><td>Yes</td><td></td></tr>
</table>

	**Was the control status established in a standard, valid and reliable way?**	Yes	
**PERFORMANCE BIAS**	**Aside from the exposure/ intervention, were the groups treated the same?**	Not reported	
	**Were outcome assessors blind to case and control status?**	Not reported	
	**Were all outcomes measured in a standard, valid and reliable way?**	Yes	The laboratory biochemical tests are standard, valid and reliable tests and the methods used are described.
	**Were outcomes assessed objectively and independently?**	Yes	
**ATTRITION BIAS**	**What percentage of the individuals recruited into each arm of the study were lost to follow up?**	Not reported	Not reported specifically, however: "Adverse effects in the total population were moderate and transient, and resolved without intervention. However, 12 women of the PCOS group and eight of the controls reported occasionally diarrhoea with faecal urgency, but none withdrew from the study."
	**What percentage of each group (cases and controls) refused to participate in the study?**	Not reported	
	**What percentage of the individuals were not included in the analysis?**	Not reported	Not reported whether analysis was made per protocol or intention to treat.

REPORT BIAS	**Is the paper free of selective outcome reporting?**	Not reported	There is no published protocol available.
CONFOUNDING	**Were the groups comparable with regards to key prognostic variables?**	Partial	At baseline the two groups differed in age, but not in BMI. However, the results are adjusted for differences in BMI.
OTHER INTERNAL VALIDITY/BIAS	**Were there any conflicts of interest in the writing or funding of this study?**	Not reported	
	**Was the study sufficiently powered to detect any differences between the groups?**	Not reported	
	**If statistical analysis was undertaken, was this appropriate?**	Yes	"Differences between groups and times of measurements (treatment effect) were investigated using linear regression models, taking into account the correlation between each subject's measurements. Specifically, we used a mixed model with random intercept and with time of measurement and group, treated as categorical variables, being the fixed effects. An interaction term between time and group was also introduced to the model. Logarithmic transformation was used when necessary (skewed distributions). The results are adjusted for differences in BMI and are presented as mean changes between groups/ times or mean relative changes if they were logarithmically

			transformed. A P-value < 0.05 was taken to indicate statistical significance.”
**Comments**			
**What is the overall risk of bias?**		High	*High - Few or no criteria fulfilled or the conclusions of the study are likely or very likely to be affected.*

Cited in full as: Monash Centre for Health Research and Implementation (MCHRI) Evidence Synthesis Program template for critical appraisal of a case control study (2014), MCHRI – Monash University and Monash Health, Melbourne, Australia (*adapted from* Critical Appraisal Templates (2010) Centre for Clinical Effectiveness, Southern Health, Melbourne, Australia AND Wells G, Shea B, O'Connell D, Peterson J, Welch V, Losos M, Tugwell P. The Newcastle–Ottawa Scale (NOS) for assessing the quality of nonrandomized studies in meta-analyses. In: Proceedings of the 3rd Symposium on Systematic Reviews Beyond the Basics: Improving Quality and Impact. Oxford; 2000. p.3-5).

Critical appraisal of a case control study

**Study ID**	Hutchison et al. 2010, Harrison et al. 2011
**Study citation**	Hutchison, S. K., et al. (2011). "Effects of exercise on insulin resistance and body composition in overweight and obese women with and without polycystic ovary syndrome." Journal of clinical endocrinology and metabolism **96**(1): E48-E56.<br><br>Harrison, C. L., et al. (2012). "The impact of intensified exercise training on insulin resistance and fitness in overweight and obese women with and without polycystic ovary syndrome." Clinical endocrinology **76**(3): 351-357.
**EXTERNAL VALIDITY – IS THIS STUDY AND ITS RESULTS GENERALIZABLE TO MY SYSTEMATIC REVIEW QUESTION?**	
**Patient/population/ participants**	Overweight and obese (BMI>27 kg/m^2) premenopausal women with PCOS, age 20-40 years (29.5±1.4).
**Control population**	Overweight and obese (BMI>27 kg/m^2) premenopausal women without PCOS age 20-40 years (35±1.1).

**N**	The number of participants that were:<br>• Screened: 117 (by phonescreening)<br>• Enrolled/eligble/started: 34 (PCOS 20 non-PCOS 14)<br>• Assessed/finished: 21 (PCOS 13 non-PCOS 8)<br>• Followed up:
**Setting**	The study was conducted at an academic medical center. Country not reported but assumed to be Australia. All authors from Australian universities.
**Intervention/indicator**	"At screening (3 months before baseline), standard diet and lifestyle advice was delivered [Heart Foundation recommendations (www.heartfoundation.org.au)] and medications affecting end points including insulin sensitizers, anti-androgens, and hormonal contraceptives were ceased. Participants undertook 12 wk of supervised intensified exercise training on a motorized treadmill (three x 1 h sessions each week) under supervision of exercise physiologists (C.L.H. and N.K.S.). One session consisted of 60 min of moderate-intensity treadmill walking/jogging that elicited work rates of 75–85% of maximal heart rate (HR^{max}) equivalent to 70% of maximal oxygen consumption ($VO2^{max}$). This alternated with high-intensity intermittent exercise, during which participants walked/ jogged on the treadmill (six x 5 min work bouts with 2 min of recovery) at an exercise intensity of 95–100% HR^{max} (equivalent to 90–100% $VO2^{max}$). Participants progressed to eight repetitions by wk 4 and reduced recovery time to 1 min by wk 8. Target exercise intensity heart rates were achieved by altering speed and incline on the treadmill according to individual fitness. $VO2^{max}$ tests were repeated at 6 wk to assess changes in fitness and HR^{max}. Heart rate monitors were used in all sessions (Polar Electro Oy, Kempele, Finland)."
**Outcomes**	• Waist circumference<br>• Weight<br>• BMI<br>• VO2max (not relevant to systematic review)<br>• Testosterone<br>• SHBG<br>• FAI<br>• Cholesterol<br>• Triglycerides<br>• HDL<br>• LDL

	• Fasting glucose<br>• Fasting insulin<br>• Fasting glucose to insulin ratio<br>• HOMA-IR<br>• Lean tissue mass<br>• Total fat mass<br>• Abdominal fat mass<br>• Visceral fat<br>• Subcutaneous fat<br>• WHR<br>• Respiratory exchange ratio (not relevant to systematic review)<br>• HR_{max} (not relevant to systematic review)<br>• HbA1c<br>• Blood pressure<br>• Impaired fasting glucose (not relevant to systematic review)	
**Does the study have a clearly focused question and/or PICO?**	Yes	
**Inclusion Criteria**	Yes	PCOS diagnosis consistent with NIH-criteria. “PCOS was diagnosed by an endocrinologist (S.K.H.) based on irregular menstrual cycles (<21 or>35 d) and clinical (hirsutism, acne) or biochemical (elevation of at least one circulating ovarian androgen) hyperandrogenism [1990 National Institutes of Health criteria]. (…)All non-PCOS women had regular menses and no evidence of clinical or biochemical hyperandrogenism.”<br><br>“All non-PCOS women had regular menses and no evidence of clinical or biochemical hyperandrogenism.”
**Exclusion Criteria**	Yes	“Hyperprolactinemia, thyroid dysfunction, and specific adrenal disorders were excluded clinically and where indicated biochemically. (…) Exclusion criteria included use of glucocorticoids, anti-hypertensives, weight loss, lipid-lowering agents, smoking, DM2, participation in regular physical activity, recent weight change, and pregnancy both at screening and during the 3-month run-in.”

**If there were specified inclusion/ exclusion criteria, were these appropriate?**		Yes	
**Is a case control study the appropriate design to answer this question?**		Yes	
**Were the outcomes measured appropriate?**		Yes	
**Was there sufficient duration of follow-up for outcomes to occur?**		Yes	
**INTERNAL VALIDITY – HAS THIS STUDY BEEN CONDUCTED RIGOROUSLY IN ORDER TO REDUCE BIAS?**			
**SELECTION BIAS**	**Were the cases and controls taken from comparable populations?**	Yes	Study participants were recruited through community advertisements.
	**Was the case definition adequate and established in a standard, valid and reliable way?**	Yes	Diagnosis according to NIH criteria.
	**Was the control status established in a standard, valid and reliable way?**	Not reported	However the inclusion critera implies that criteria were applied given that all non-PCOS needed to have regular menses and no evidence of clinical or biochemical hyperandrogenism.
**PERFORMANC E BIAS**	**Aside from the exposure/ intervention, were the groups treated the same?**	Not reported	

	**Were outcome assessors blind to case and control status?**	Not reported	For some outcomes it won't result in bias, but some subjective outcomes are at risk of bias, such as waist, body composition, VF and SCFAT. However for the VF and SCFAT intra-reader variability has been done.<br><br>Abdominal VF and SCFAT were assessed with participants placed supine with arms extended above their head. And therefore there's the potential for inconsistency in level of extention of the arms.
	**Were all outcomes measured in a standard, valid and reliable way?**	Yes	The laboratory biochemical tests are standard, valid and reliable tests and the methods used are described.
	**Were outcomes assessed objectively and independently?**	Yes	
ATTRITION BIAS	**What percentage of the individuals recruited into each arm of the study were lost to completion of study?**	35 % PCOS<br>43 % control/ comparison	7/20 PCOS did not complete the study.<br>6/14 non-PCOS did not complete the study.<br><br>13 patients did not complete due to lost to contact (n=4 PCOS), discontinued intervention (n=3 PCOS, 5 non-PCOS), protocol violation (n=1 non-PCOS commenced significant sustained physical activity).<br>No comparisons were made between participants followed-up and those lost to follow up.
	**What percentage of each group (cases and controls) refused to participate in the study?**	15% PCOS<br>36% control	3/20 PCOS and 5/14 non-PCOS refused to participate in the study.
	**What percentage of the individuals**	35 % PCOS<br>43% control/ comparison	7/20 PCOS and 6/14 non-PCOS were not included in the analysis.

	**were not included in the analysis?**		Not reported whether analysis was made per protocol or intention to treat.
REPORT BIAS	**Is the paper free of selective outcome reporting?**	Not reported	There is no published protocol available.
CONFOUNDING	**Were the groups comparable with regards to key prognostic variables?**	Yes	Groups were not comparable for age but were comparable for BMI.<br>Data were log transformed if not normally distributed (insulin, HOMA) and assessed using Student's t test with general linear modeling to correct for age.
OTHER INTERNAL VALIDITY/BIAS	**Were there any conflicts of interest in the writing or funding of this study?**	No	
	**Was the study sufficiently powered to detect any differences between the groups?**	No	"Power calculations, based on a previous non-PCOS exercise<br>study demonstrating a decrease in IR and a decrease in VF,<br>suggested that the current study has a power of 80% and an α-level 0.05 with a required sample size of 7."<br><br>Power calculation for only one to us relevant variable, IR. For that an adequate sample size was undertaken.
	**If statistical analysis was undertaken, was this appropriate?**	Yes	"Two-tailed statistical analysis was performed using SPSS for Windows 17.0 software (SPSS Inc., Chicago, IL) with statistical significance set at α-level of $P<0.05$. Data were log transformed if not normally distributed (insulin, HOMA) and assessed using Student's t test with general linear modeling to correct for age. The effect of exercise was assessed

			using repeated-measures ANOVA with PCOS status as between-subject factor and exercise as within-subject factor. Relationships between variables were examined using bivariate (Pearson) correlations and the impact of covariates assessed using linear regression. Change in variable was defined as the percentage difference between pre- and post-training values."
**Comments**			
**What is the overall risk of bias?**		Moderate	*Moderate - Some of the criteria have been fulfilled and those criteria that have not been fulfilled may affect the conclusions of the study.*

Cited in full as: Monash Centre for Health Research and Implementation (MCHRI) Evidence Synthesis Program template for critical appraisal of a case control study (2014), MCHRI – Monash University and Monash Health, Melbourne, Australia (*adapted from* Critical Appraisal Templates (2010) Centre for Clinical Effectiveness, Southern Health, Melbourne, Australia AND Wells G, Shea B, O'Connell D, Peterson J, Welch V, Losos M, Tugwell P. The Newcastle–Ottawa Scale (NOS) for assessing the quality of nonrandomized studies in meta-analyses. In: Proceedings of the 3rd Symposium on Systematic Reviews Beyond the Basics: Improving Quality and Impact. Oxford; 2000. p. 3-5).

Critical appraisal of a case control study

**Study ID**	Kowalska 2001
**Study citation**	Kowalska, I., et al. (2001). "Insulin, leptin, IGF-I and insulin-dependent protein concentrations after insulin-sensitizing therapy in obese women with polycystic ovary syndrome." European Journal of Endocrinology 144(5): 509-515.
**EXTERNAL VALIDITY – IS THIS STUDY AND ITS RESULTS GENERALIZABLE TO MY SYSTEMATIC REVIEW QUESTION?**	
**Patient/population/ participants**	Obese women with PCOS age 25.4±4.8, BMI 34.7±6.0. Obesity was defined as a body mass index (BMI) of more than 27.5 kg/m^2.
**Control population**	Obese women without PCOS age 27.9±7.3, BMI 36.2±6.0.

	Obesity was defined as a body mass index (BMI) of more than 27.5 kg/m^2.<br>Non-obese women without PCOS age 30.4±5.7. BMI 21.9±2.0.	
**N**	The number of participants that were:<br>• Screened: (baseline) 53 (23 obese PCOS, 19 obese non-PCOS, 11 control)<br>• Enrolled/started: 25 (15 obese PCOS, 10 obese non-PCOS)<br>• Assessed: 17 (11 obese PCOS, 6 obese non-PCOS)<br>• Followed up: -	
**Setting**	Department of Endocrinology and Endocrine Gynecology, Medical Academy, Bialystok, Poland.	
**Intervention/indicator**	"Patients were administered a hypocaloric diet (1200-1400 kcal/24 h) and metformin (Polfa, Kutno, Poland) therapy (500 mg three times a day) for a period of 4-5 months. After 4-5 months of therapy all pre-treatment studies were repeated."	
**Outcomes**	• BMI<br>• % body fat<br>• Waist circumference<br>• Hip circumference<br>• WHR<br>• Testosterone<br>• FAI<br>• LH (not relevant to systematic review)<br>• FSH (not relevant to systematic review)<br>• LH/FSH (not relevant to systematic review)<br>• Oestradiol (not relevant to systematic review)<br>• SHBG<br>• IGF-1 (not relevant to systematic review)<br>• IGF-BP1 (not relevant to systematic review)<br>• Leptin (not relevant to systematic review)<br>• Fasting insulin	
**Does the study have a clearly focused question and/or PICO?**	Partial	"The aim of this study was to test the hypothesis that metformin and hypocaloric diet improves insulin sensitivity in obese PCOS women through its influence on insulin and glucose concentrations, insulin-dependent protein concentrations (sex hormone-binding protein (SHBG) and insulin-like growth factor-binding protein-1 (IGFBP-1)) and insulin-like growth factor-I (IGF-I)."

		No comparison was given.
**Inclusion Criteria**	Yes	"The diagnosis of PCOS was made according to the characteristic clinical findings (the presence of oligo/amenorrhea and hirsutism), laboratory data (testosterone concentrations elevated or in the upper limit of normal) and all patients had polycystic ovaries shown by transvaginal ultrasonography (>8 subcapsular follicles of 3-8 mm diameter in one plane in one ovary and increased stroma). We considered that patients had oligomenorrhea if they had fewer than six menstrual periods in the preceding year. Amenorrhea was considered as the absence of periods for >6 months. Hirsutism was evaluated using Ferriman-Gallwey scoring system, before the study.<br><br>The patient was described as hirsute if the score was more than 10. Testosterone concentrations were determined in the loca laboratory using chemiluminescence immunoassay. The range of normal values is from 0.2 to 0.8 ng/ml. We considered that a patient had elevated testosterone concentrations if the concentration exceeded 0.8 ng/ml (19/23 patients: 83% of studied group).<br><br>A patient was included in the PCOS group if she had ultrasound features of PCOS and fulfilled at least two of the following criteria: oligomenorrhea/amenorrhea, hirsutism and serum androgens in the upper limit of normal or elevated."<br><br>Obese women and the control group had regular menstrual cycles.
**Exclusion Criteria**	Yes	"Other reasons for menstrual disturbances (non-classical 21-hydroxylase deficiency, hyperprolactinemia, androgen-secreting tumors and thyroid dysfunction) were excluded by appropriate tests before the study. None of the women was on a diet program or had been taking any drug known to affect carbohydrate

			metabolism for at least 2 months prior to the metabolic and endocrine investigations.”
**If there were specified inclusion/ exclusion criteria, were these appropriate?**		Yes	
**Is a case control study the appropriate design to answer this question?**		Yes	
**Were the outcomes measured appropriate?**		Yes	
**Was there sufficient duration of follow-up for outcomes to occur?**		Yes	
**INTERNAL VALIDITY – HAS THIS STUDY BEEN CONDUCTED RIGOROUSLY IN ORDER TO REDUCE BIAS?**			
**SELECTION BIAS**	**Were the cases and controls taken from comparable populations?**	Not reported	
	**Was the case definition adequate and established in a standard, valid and reliable way?**	Yes	
	**Was the control status established in a standard, valid and reliable way?**	Not reported	Not reported, however it is reported that obese women and the control group had regular menstrual cycles. But not reported if any diagnostic criteria had been performed on these women.

Cited in full as: Monash Centre for Health Research and Implementation (MCHRI) Evidence Synthesis Program template for critical appraisal of a case control study (2014), MCHRI – Monash University and Monash Health, Melbourne, Australia (*adapted from* Critical Appraisal Templates (2010) Centre for Clinical Effectiveness, Southern Health,

PERFORMANCE BIAS	**Aside from the exposure/ intervention, were the groups treated the same?**	Not reported	
	**Were outcome assessors blind to case and control status?**	Not reported	
	**Were all outcomes measured in a standard, valid and reliable way?**	Yes	The laboratory biochemical tests are standard, valid and reliable tests and the methods used are described.
	**Were outcomes assessed objectively and independently?**	Yes	BMI, WHR and FAI were calculated, but not reported in what way.
ATTRITION BIAS	**What percentage of the individuals recruited into each arm of the study were lost to follow up?**	27% treatment<br>40% control/ comparison	4/ 15 taking metformin in the PCOS-obese group and<br>4/10 patients from the obese regularly menstruating women did not complete the study.<br>In the PCOS-obese group 2 patients conceived and delivered healthy children at term, 2 patients discontinued the study because of mild gastrointestinal side effects.<br>In the group of obese patients without menstrual disturbances 4 patients discontinued the study due to mild gastrointestinal side effects (nausea, diarrhea).
	**What percentage of each group (cases and controls) refused to participate in the study?**	Not reported	A large difference in participation rate between the cases and control may indicate that a significant degree of selection bias may be present, and the study results should be treated with considerable caution.<br>Consider if comparisons were made between participants and non-participants to establish their similarities or differences.<br>Even if participation rates are comparable and acceptable, it is still possible that the

			participants selected to act as cases or controls may differ from other members of the source population in some significant way. A well conducted case-control study will look at samples of the non-participants among the source population to ensure that the participants are a truly representative sample
	**What percentage of the individuals were not included in the analysis?**	27% treatment<br>40 % control/ comparison	Not reported whether analysis was made per protocol or intention to treat.
**REPORT**	**Is the paper free of selective outcome reporting?**	Not reported	There is no published protocol available
**CONFOUNDING**	**Were the groups comparable with regards to key prognostic variables?**	Yes	Groups were comparable to age and BMI. No matching was done. Economic status is not considered to be relevant here.
**OTHER INTERNAL VALIDITY/BIAS**	**Were there any conflicts of interest in the writing or funding of this study?**	Not reported	
	**Was the study sufficiently powered to detect any differences between the groups?**	Not reported	

Melbourne, Australia AND Wells G, Shea B, O'Connell D, Peterson J, Welch V, Losos M, Tugwell P. The Newcastle–Ottawa Scale (NOS) for assessing the quality of nonrandomized

	**If statistical analysis was undertaken, was this appropriate?**	Yes	"The pre- and post-treatment data within the groups were compared using Wilcoxon rank sum test. The results between the groups were analyzed using the Mann±Whitney U test. Correlations were estimated using simple regression analysis. Data are expressed as mean ±SD and p <0.05 was considered statistically significant."
**Comments**			
**What is the overall risk of bias?**		High	*High - Few or no criteria fulfilled or the conclusions of the study are likely or very likely to be affected.*

studies in meta-analyses. In: Proceedings of the 3rd Symposium on Systematic Reviews Beyond the Basics: Improving Quality and Impact. Oxford; 2000. p. 3-5).

Critical appraisal of a case control study

**Study ID**	Moran 2007
**Study citation**	Moran, L. J., et al. (2007). "C-reactive protein before and after weight loss in overweight women with and without polycystic ovary syndrome." Journal of Clinical Endocrinology & Metabolism **92**(8): 2944-2951.
**EXTERNAL VALIDITY – IS THIS STUDY AND ITS RESULTS GENERALIZABLE TO MY SYSTEMATIC REVIEW QUESTION?**	
**Patient/population/ participants**	Overweight premenopausal women (European Caucasian) with PCOS, age 31.7±6.2, weight 95.1±19.3, BMI 35.7±5.8.
**Control population**	Overweight premenopausal women without PCOS, age 37.1±4.7, weight 95.5±16.5, BMI 35.5±5.1.
**N**	The number of participants that were:<br>• Screened: 62<br>• Enrolled: 37 (18 PCOS 19 non-PCOS)<br>• Assessed: 32 (15 PCOS 17 non-PCOS)<br>• Followed up: -
**Setting**	The study was conducted on an outpatient basis at a clinic. Country not reported but assumed to be Australia. All authors from Australian universities.

Cited in full as: Monash Centre for Health Research and Implementation (MCHRI) Evidence Synthesis Program template for critical appraisal of a case control study (2014),

**Intervention/indicator**	"Subjects followed an energy-restricted diet whereby two meals daily were re- placed with commercially available meal replacements (Slimfast; Unilever Australasia, Epping, New South Wales, Australia) for 8 weeks."	
**Outcomes**	• Weight<br>• Waist circumference<br>• Total fat mass<br>• Total fat free mass<br>• Total cholesterol<br>• LDL<br>• HDL<br>• Triglycerides<br>• Adiponectin (not relevant to systematic review)<br>• IL-6<br>• TNF-α<br>• Insulin<br>• HOMA-IR<br>• Testosterone<br>• SHBG<br>• FAI<br>• Free testosterone	
**Does the study have a clearly focused question and/or PICO?**	Yes	
**Inclusion Criteria**	Yes	PCOS was diagnosed according to Rotterdam consensus group criteria. No information available if diagnostic criteria were applied to controls.
**Exclusion Criteria**	Yes	"Exclusion criteria were pregnancy, breastfeeding, body mass index (BMI) less than 25 kg/m2, and use of oral contraceptives, endocrine hormonal treatment, or insulin-sensitizing agents (subjects were required to cease oral contraceptives 4 wk and hormonal treatment/insulin-sensitizing agents 2wk before commencement of the study)."
**If there were specified inclusion/ exclusion**	Yes	

criteria, were these appropriate?			
Is a case control study the appropriate design to answer this question?		Yes	
Were the outcomes measured appropriate?		Yes	
Was there sufficient duration of follow-up for outcomes to occur?		Yes	
**INTERNAL VALIDITY – HAS THIS STUDY BEEN CONDUCTED RIGOROUSLY IN ORDER TO REDUCE BIAS?**			
**SELECTION BIAS**	**Were the cases and controls taken from comparable populations?**	Yes	Study participants were recruited through community advertisements.
	**Was the case definition adequate and established in a standard, valid and reliable way?**	Yes	
	**Was the control status established in a standard, valid and reliable way?**	Not reported	No information whether PCOS diagnostic criteria were applied on controls.
**PERFORMANCE BIAS**	**Aside from the exposure/ intervention, were the groups treated the same?**	Not reported	
	**Were outcome assessors blind to**	Not reported	

	**case and control status?**		
	**Were all outcomes measured in a standard, valid and reliable way?**	Yes	The laboratory biochemical tests are standard, valid and reliable tests and the methods used are described.
	**Were outcomes assessed objectively and independently?**	Yes	The homeostatic model assessment (HOMA) was used as a surrogate measure of insulin sensitivity, calculated as [fasting insulin (mU/liter) x fasting glucose (mmol/liter)/22.5]. The free androgen index (FAI) (testosterone/SHBGx100) and equilibrium binding equations for determination of free testosterone were used as surrogate estimates of free testosterone.
**ATTRITION BIAS**	**What percentage of the individuals recruited into each arm of the study were lost to follow up?**	17 % treatment<br>11% control/ comparison	3/18 PCOS and 2/19 non-PCOS were lost to completion due to discontinued intervention.
	**What percentage of each group (cases and controls) refused to participate in the study?**	Not reported	
	**What percentage of the individuals were not included in the analysis?**	17% treatment<br>11% control/ comparison	3/18 PCOS and 2/19 non-PCOS were not included in the analysis.<br>Not reported whether analysis was made per protocol or intention to treat.
**REPORT**	**Is the paper free of selective outcome reporting?**	Not reported	There is no published protocol available.

CONFOUNDING	**Were the groups comparable with regards to key prognostic variables?**	Yes	Groups differed in age at baseline, but were comparable regarding BMI. Groups were matched for BMI and smoking status.
OTHER INTERNAL VALIDITY/BIAS	**Were there any conflicts of interest in the writing or funding of this study?**	Not reported	
	**Was the study sufficiently powered to detect any differences between the groups?**	No	"This study had 65% power to detect a difference of 1.6 mg/liter between subjects with and without PCOS for CRP to statistical significance of P < 0.05. To confirm the observed differences between subjects with and without PCOS of CRP to statistical significance of P < 0.05 and 80% power, 19 subjects for each group would be needed. For changes in adiponectin with weight loss, 124 subjects would be needed in each group to detect a difference of 633.4 ng/ml between the subjects with and without PCOS to statistical significance of P <0.05 and 80% power.<br><br>Power calculation for two variables, CRP and adiponectin. For no one of these was an adequate sample size undertaken.
	**If statistical analysis was undertaken, was this appropriate?**	Yes	Data were presented as means ± sd except where indicated and log transformed where non-normally distributed.<br>Two-tailed statistical analysis was performed using SPSS for Windows 14.0 software (SPSS Inc., Chicago, IL) with statistical significance set at an α-level of P < 0.05. Baseline data were assessed using a one-way ANOVA. Comparisons between time points were

			assessed using repeated-measures ANOVA with PCOS as between-subject factor. In specific analyses, weight, BMI, age, and weight loss were included as covariates. In the event of an interaction, post hoc pairwise comparisons were performed. Relationships between variables were examined using bivariate and partial correlations. Subjects with baseline CRP above and below the median (4.53 mg/liter) were assessed separately with baseline CRP status as the between-subject factor.
**Comments**			
**What is the overall risk of bias?**		Moderate	*Moderate - Some of the criteria have been fulfilled and those criteria that have not been fulfilled may affect the conclusions of the study.*

Melbourne, Australia AND Wells G, Shea B, O'Connell D, Peterson J, Welch V, Losos M, Tugwell P. The Newcastle–Ottawa Scale (NOS) for assessing the quality of nonrandomized studies in meta-analyses. In: Proceedings of the 3rd Symposium on Systematic Reviews Beyond the Basics: Improving Quality and Impact. Oxford; 2000. p. 3-5).

Critical appraisal of a case control study

**Study ID**	Panidis et al. 2014, Vosnakis et al. 2013, Panidis et al. 2008
**Study citation**	Panidis, D., et al. (2014). "The role of orlistat combined with lifestyle changes in the management of overweight and obese patients with polycystic ovary syndrome." Clinical endocrinology 80(3): 432-438.<br><br>Vosnakis, C., et al. (2013). "Diet, physical exercise and Orlistat administration increase serum anti-Mullerian hormone (AMH) levels in women with polycystic ovary syndrome (PCOS)." Gynecological endocrinology **29**(3): 242-245.<br><br>Panidis, D., et al. (2008). "Obesity, weight loss, and the polycystic ovary syndrome: effect of treatment with diet and orlistat for 24 weeks on insulin resistance and androgen levels." Fertility and sterility **89**(4): 899-906.

EXTERNAL VALIDITY – IS THIS STUDY AND ITS RESULTS GENERALIZABLE TO MY SYSTEMATIC REVIEW QUESTION?	
**Patient/population/ participants**	Women with PCOS age 26,1±6,4 years, BMI 34,5±5,9 kg/m^2.
**Control population**	Women without PCOS age 31,5±4,7 years, BMI 34,9±5,4 kg/m^2.
**N**	The number of participants that were:<br>• Screened: -<br>• Enrolled: 101 PCOS 29 controls<br>• Assessed: 101 PCOS 29 controls<br>• Followed up: -
**Setting**	Gynecological Endocrinology<br>Infirmary of the Second Department of Obstetrics and Gynecology, Aristotle University of Thessaloniki.
**Intervention/indicator**	"All women were prescribed a normal-protein, energy restricted diet (basic metabolic rate – 600 kcal/day, consisting of 50% from carbohydrate, 30% from fat (10% saturated) and 20% from protein) and were instructed to exercise 3 days a week for<br>1 h for a period of 6 months. Moderate intensity, aerobic exercise (e.g. brisk walking) was advised. Participants were educated and given diet and exercise advice at one session. No nutritionists or exercise specialists were involved in the study. All women were also given orlistat 120 mg t.i.d before each meal for 6 months [Xenical, Roche (Hellas) S.A., Greece]."
**Outcomes**	• Weight –no numbers presented<br>• Waist circumference–no numbers presented (Panidis 2008 presented for 18 PCOS & 14 controls)<br>• BMI–no numbers presented (Vosnakis presented for 61 PCOS & 20 controls)<br>• WHR- (Vosnakis presented for 61 PCOS & 20 controls)<br>• Total cholesterol<br>• LDL<br>• HDL<br>• Triglycerides<br>• Glucose<br>• Insulin<br>• Glucose/insulin<br>• AUC-OGTT<br>• HOMA-IR<br>• QUICKI (not relevant to systematic review)

	• FSH (not relevant to systematic review)<br>• LH (not relevant to systematic review)<br>• Prolactin (not relevant to systematic review)<br>• Testosterone<br>• Δ_4-A (not relevant to systematic review)<br>• DHEA-S (not relevant to systematic review)<br>• FAI<br>• 17α-OHP (not relevant to systematic review)<br>• SHBG<br>• Ovarian volume (not relevant to systematic review)<br>• Ovarian follicles (not relevant to systematic review)	
**Does the study have a clearly focused question and/or PICO?**	Yes	
**Inclusion Criteria**	Yes	Diagnosis of PCOS was based on the revised criteria of Rotterdam.<br><br>Controls were women with normal ovulating cycles (duration 28±2 days, serum progesterone levels >10 ng/ml in two consecutive cycles), without clinical or biochemical signs of hyperandrogenism and without polycystic ovaries on ultrasound.
**Exclusion Criteria**	Yes	"None of the studied women had galactorrhoea or any endocrine or systemic disease that could possibly affect reproductive physiology. A Synachten test was performed with tetracosactide (Synachten 0.25 mg/1 ml; Novartis Pharma, Rueil-Malmaison, France) in women with basal plasma 17α-hydroxyprogesterone (17α-OHP) levels >1.5 ng/ml to exclude congenital adrenal hyperplasia. No woman reported use of any medication that could interfere with the normal function of the hypothalamic–pituitary–gonadal axis (including metformin and oral contraceptives) during the last semester. Except orlistat, no other medication was administered during the study period of 6 months."
**If there were specified inclusion/ exclusion**	Yes	

criteria, were these appropriate?			
Is a case control study the appropriate design to answer this question?		Yes	
Were the outcomes measured appropriate?		Yes	
Was there sufficient duration of follow-up for outcomes to occur?		Yes	
**INTERNAL VALIDITY – HAS THIS STUDY BEEN CONDUCTED RIGOROUSLY IN ORDER TO REDUCE BIAS?**			
SELECTION BIAS	Were the cases and controls taken from comparable populations?	Not reported	
	Was the case definition adequate and established in a standard, valid and reliable way?	Yes	
	Was the control status established in a standard, valid and reliable way?	Yes	
PERFORMANCE BIAS	Aside from the exposure/ intervention, were the groups treated the same?	Not reported	
	Were outcome assessors blind to	Not reported	

	**case and control status?**		
	**Were all outcomes measured in a standard, valid and reliable way?**	Yes	The laboratory biochemical tests are standard, valid and reliable tests and the methods used are described.
	**Were outcomes assessed objectively and independently?**	Yes	BMI was calculated by dividing weight (in kg) by height squared (in m) to assess obesity. WHR was calculated by dividing W by H.<br><br>Free androgen index (FAI) was determined as follows: FAI = T (nmol/l) x100/SHBG (nmol/l).<br>The homeostasis model assessment of insulin resistance<br>(HOMA-IR) index was calculated as follows: HOMA-IR = fasting<br>insulin (μIU/ml) x fasting glucose (mg/dl)/405. The quantitative<br>insulin sensitivity check index (QUICKI) was calculated<br>according to the following formula: QUICKI = 1/[logInsulin<br>(lIU/ml) + logGlucose (mg/dl)].
**ATTRITION BIAS**	**What percentage of the individuals recruited into each arm of the study were lost to follow up?**	Not reported	
	**What percentage of each group (cases and controls) refused to participate in the study?**	Not reported	Participation rate is not reported. No comparisons were made between participants and non-participants to establish their similarities or differences.
	**What percentage of the individuals were not included in the analysis?**	Not reported	

REPORT BIAS	**Is the paper free of selective outcome reporting?**	Not reported	No published protocol available.
CONFOUNDING	**Were the groups comparable with regards to key prognostic variables?**	Partial	Groups were matched for BMI. There was significant difference in age. Economic status is not reported, but is not relevant here.
OTHER INTERNAL VALIDITY/BIAS	**Were there any conflicts of interest in the writing or funding of this study?**	No	
	**Was the study sufficiently powered to detect any differences between the groups?**	Not reported	
	**If statistical analysis was undertaken, was this appropriate?**	Yes	"Data analysis was performed with the statistical package SPSS (version 17.0; SPSS Inc., Chicago, IL, USA). Results are reported as mean ±SD. Changes between baseline and end-of-treatment were assessed with two-way repeated measures analysis of variance. Correlations between changes in BMI and changes in other parameters were assessed with Pearson correlation. In all cases, a P value <0.05 was considered significant."
**Comments**		We have looked at data mainly from Panidis 2014 where they included data from two previous studies done by this group (Vosnakis 2012, Panidis 2008) using the same methodology. Where sought outcomes weren´t found in	

	Panidis 2014, they were extracted from the two other studies.<br>They report: "Women with PCOS and controls who were studied in previous smaller reports on the effects of orlistat from the same group were included in this study." Risk of bias is done based on all three articles.	
**What is the overall risk of bias?**	High	*High - Few or no criteria fulfilled or the conclusions of the study are likely or very likely to be affected.*

Cited in full as: Monash Centre for Health Research and Implementation (MCHRI) Evidence Synthesis Program template for critical appraisal of a case control study (2014), MCHRI – Monash University and Monash Health, Melbourne, Australia (*adapted from* Critical Appraisal Templates (2010) Centre for Clinical Effectiveness, Southern Health, Melbourne, Australia AND Wells G, Shea B, O'Connell D, Peterson J, Welch V, Losos M, Tugwell P. The Newcastle–Ottawa Scale (NOS) for assessing the quality of nonrandomized studies in meta-analyses. In: Proceedings of the 3rd Symposium on Systematic Reviews Beyond the Basics: Improving Quality and Impact. Oxford; 2000. p. 3-5).

Critical appraisal of a case control study

**Study ID**	Pasquali 2000
**Study citation**	Pasquali, R., et al. (2000). "Effect of long-term treatment with metformin added to hypocaloric diet on body composition, fat distribution, and androgen and insulin levels in abdominally obese women with and without the polycystic ovary syndrome." Journal of clinical endocrinology and metabolism **85**(8): 2767-2774.
**EXTERNAL VALIDITY – IS THIS STUDY AND ITS RESULTS GENERALIZABLE TO MY SYSTEMATIC REVIEW QUESTION?**	
**Patient/population/ participants**	Obese women with PCOS with body mass index (BMI) values greater than 28, and abdominal body fat distribution defined by waist-to-hip ratio (WHR) values greater than 0.80.
**Control population**	Obese women without PCOS with body mass index (BMI) values greater than 28, and abdominal body fat distribution defined by waist-to-hip ratio (WHR) values greater than 0.80.
**N**	The number of participants that were:<br>• Screened: -

	• Enrolled: 40 (20 PCOS, 20 controls). Metformin (12 PCOS, 8 controls), Placebo (8 PCOS, 12 controls)<br>• Assessed: 35 (18 PCOS, 17 controls). Metformin (10 PCOS, 8 controls), Placebo (8 PCOS, 9 controls)<br>• Followed up: -	
**Setting**	Endocrine Unit of the Department of Internal Medicine and Gastroenterology of the Orsola-Malpighi Hospital of Bologna.	
**Intervention/indicator**	"All women were placed, for a month, on a standardized hypocaloric diet consisting of 1200–1400 kcal daily and containing 50% carbohydrates, 30%total lipids and 20% proteins. The women returned after 1 month and while continuing dietary treatment, PCOS women and obese controls were subsequently placed, in a random order, on metformin 850 mg/os, twice daily or for the following 6 months."	
**Outcomes**	• Weight<br>• BMI<br>• Waist circumference<br>• Hip circumference<br>• WHR<br>• TAT<br>• SAT<br>• VAT<br>• VAT/SAT<br>• Fasting glucose<br>• $Glucose_{AUC}$ (not relevant to systematic review)<br>• Fasting insulin<br>• $Insulin_{AUC}$ (not relevant to systematic review)<br>• Fasting C-peptide (not relevant to systematic review)<br>• $C\text{-}peptide_{AUC}$ (not relevant to systematic review)<br>• LH (not relevant to systematic review)<br>• FSH (not relevant to systematic review)<br>• Testosterone<br>• DHEA-S (not relevant to systematic review)<br>• E2 (not relevant to systematic review)<br>• SHBG	
**Does the study have a clearly focused question and/or PICO?**	Yes	
**Inclusion Criteria**	Yes	The diagnosis of PCOS was made according to the presence of oligomenorrhea (less than four cycles in the last 6 months) or amenorrhea (no menses in the last 6 months) and hyperandrogenism, defined

		by supranormal total and free T concentrations, according to normal reference values in our laboratory. All women women with PCOS had ovarian ultrasonic findings consistent with the diagnosis.<br><br>Women of the control group had regular monthly menses and no clinically or laboratory evidence of androgen excess.
**Exclusion Criteria**	Yes	None of the PCOS or control women had thyroid dysfunction, type II diabetes, or concomitant cardiovascular, renal, and liver dysfunction, based on clinical examination and routine laboratory findings.<br><br>Other causes of hyperandrogenisms, such as Cushing syndrome and disease and congenital adrenal hyperplasia, were excluded.<br><br>All PCOS women also had normal prolactin levels.<br><br>None of the PCOS or control women had taken any medication for at least 3 months before the study, nor were they dieting.
**If there were specified inclusion/ exclusion criteria, were these appropriate?**	Yes	
**Is a case control study the appropriate design to answer this question?**	Yes	
**Were the outcomes measured appropriate?**	Yes	
**Was there sufficient duration of follow-up for outcomes to occur?**	Yes	

**INTERNAL VALIDITY – HAS THIS STUDY BEEN CONDUCTED RIGOROUSLY IN ORDER TO REDUCE BIAS?**			
**SELECTION BIAS**	**Were the cases and controls taken from comparable populations?**	Yes	Patients were recruited as outpatients attending the Endocrine Unit of the Department of Internal Medicine and Gastroenetrology at S.Orsola-Malpighi Hospital of Bologna.
	**Was the case definition adequate and established in a standard, valid and reliable way?**	Yes	
	**Was the control status established in a standard, valid and reliable way?**	Yes	
**PERFORMANC E BIAS**	**Aside from the exposure/ intervention, were the groups treated the same?**	Not reported	
	**Were outcome assessors blind to case and control status?**	Not reported	
	**Were all outcomes measured in a standard, valid and reliable way?**	Yes	The laboratory biochemical tests are standard, valid and reliable tests and the methods used are described.

	**Were outcomes assessed objectively and independently?**	Yes	BMI, WHR, VAT/SAT were the only outcomes that were not independent.
**ATTRITION BIAS**	**What percentage of the individuals recruited into each arm of the study were lost to follow up?**	Placebo<br>0 % PCOS<br>25% controls<br><br>Metformin<br>17% PCOS<br>0% control	Diet+Placebo<br>PCOS: 0/8<br>Control: 3/12<br>(3 controls excluded because of non-compliance with diet)<br><br>Diet+Metformin<br>PCOS: 2/12<br>Control: 0/8<br>(2 PCOS excluded because they became pregnant)
	**What percentage of each group (cases and controls) refused to participate in the study?**	Not reported	
	**What percentage of the individuals were not included in the analysis?**	Placebo<br>0 % PCOS<br>25% controls<br><br>Metformin<br>17% PCOS<br>0% control	
**REPORT BIAS**	**Is the paper free of selective outcome reporting?**	Not reported	No published protocol available.
**CONFOUNDING**	**Were the groups comparable with regards to key prognostic variables?**	Yes	Groups were comparable regarding age and BMI.

Cited in full as: Monash Centre for Health Research and Implementation (MCHRI) Evidence Synthesis Program template for critical appraisal of a case control study (2014), MCHRI – Monash University and Monash Health, Melbourne, Australia (*adapted from* Critical Appraisal Templates (2010) Centre for Clinical Effectiveness, Southern Health,

OTHER INTERNAL VALIDITY/BIAS			
	**Were there any conflicts of interest in the writing or funding of this study?**	Not reported	
	**Was the study sufficiently powered to detect any differences between the groups?**	Not reported	
	**If statistical analysis was undertaken, was this appropriate?**	Yes	"Results are reported as the mean values ±sd, unless otherwise indicated. The response of glucose, insulin, and C-peptide to the OGTT was analyzed by calculating the (AUC) by the trapezoidal method. Normal distribution and homoscedasticity of continuous variables were tested by means of the Kolmogorov-Sminorv and the Levene tests. Variables that did not fulfill these tests were log transformed before analysis. To avoid multiple comparisons, the data at the different times of the study were evaluated by means of two-way ANOVA, applying a within-treatment and group design, while the within-subject ANOVA, with the same design, was used to compare the modifications observed during the course of the study. The scores of clinical parameters were analyzed by means of the Wilcoxon matched-pairs and the Mann-Whitney tests. Statistical evaluations were performed by running the SPSS, Inc. (Chicago, IL)/PC1 software package on a personal computer. Two-tailed P values less than 0.05 were used to define statistical significance."
**Comments**			

What is the overall risk of bias?	Moderate	*Moderate - Some of the criteria have been fulfilled and those criteria that have not been fulfilled may affect the conclusions of the study.*

Melbourne, Australia AND Wells G, Shea B, O'Connell D, Peterson J, Welch V, Losos M, Tugwell P. The Newcastle–Ottawa Scale (NOS) for assessing the quality of nonrandomized studies in meta-analyses. In: Proceedings of the 3rd Symposium on Systematic Reviews Beyond the Basics: Improving Quality and Impact. Oxford; 2000. p. 3-5).

Critical appraisal of a case control study

**Study ID**	Toscani 2011
**Study citation**	Toscani, M. K., et al. (2011). "Effect of high-protein or normal-protein diet on weight loss, body composition, hormone, and metabolic profile in southern Brazilian women with polycystic ovary syndrome: a randomized study." Gynecological endocrinology **27**(11): 925-930.
**EXTERNAL VALIDITY – IS THIS STUDY AND ITS RESULTS GENERALIZABLE TO MY SYSTEMATIC REVIEW QUESTION?**	
**Patient/population/ participants**	Women with PCOS age 22.72+5.68 years. Most study participants had BMI ≥25 kg/m^2. Fifteen of 18 (83.3%) patients with PCOS were Caucasian. The remaining participants were of mixed (African and European) descent.
**Control population**	Women without PCOS age 29.35+5.74 years. Most study participants had BMI ≥25 kg/m^2. 16 of 23 (69.6%) controls were Caucasian. The remaining participants were of mixed (African and European) descent.
**N**	The number of participants that were:<br>• Screened: 99<br>• Enrolled: 40 (18 PCOS 22 controls)<br>• Assessed: 18 PCOS 22 controls<br>• Followed up: -
**Setting**	University Hospital. Country not reported but assumed to be Brazil. All authors from Brazilian universities.
**Intervention/indicator**	"Energy needs were estimated by using 20–25 kcal/kg current weight/day for overweight/obese women and 25–30 kcal/kg current weight/day for normoweight participants. Patients were randomized to receive one of two diets: HP (30% protein, 40% carbohydrate, and 30% lipid) or NP (15% protein, 55% carbohydrate, and

	30% lipid).” 2 months of HP or NP diet.	
**Outcomes**	• Weight<br>• Waist circumference<br>• Physical activity (number of steps) (not relevant to systematic review)<br>• Systolic BP<br>• Diastolic BP<br>• Fasting glucose<br>• 2 hour glucose<br>• Fasting insulin<br>• 2 hour insulin<br>• HOMA-IR<br>• Total cholesterol<br>• HDL<br>• LDL<br>• NHDL (not relevant to systematic review)<br>• Triglycerides<br>• Total testosterone (result only in graphs)<br>• SHBG (result only in graphs)<br>• FAI (result only in graphs)<br>• BMI (result only in graphs)<br>• % Body fat (result only in graphs)<br>• Sum of trunk skinfolds (not relevant to systematic review)	
**Does the study have a clearly focused question and/or PICO?**	Yes	
**Inclusion Criteria**	Yes	“BMI ranging from 18.5 to 39.9 kg/m^2 and age between 14 and 35 years. PCOS was considered in hirsute women presenting oligo/ amenorrheic cycles (9 or less cycles/year), increased testosterone levels and/or free androgen index, and absence of other disorders causing hirsutism with or without polycystic ovaries at ultrasound. A control group was set up with BMI-matched non-hirsute women with ovulatory cycles (mid-luteal progesterone 43.8<br>ng/ml).”
**Exclusion Criteria**	Yes	“Women who had received any drugs known to interfere with hormone levels for at least 3 months before the study, with diabetes, liver or renal disease, or thyroid dysfunction were excluded from the study.”

**If there were specified inclusion/ exclusion criteria, were these appropriate?**		Yes	
**Is a case control study the appropriate design to answer this question?**		Yes	
**Were the outcomes measured appropriate?**		Yes	
**Was there sufficient duration of follow-up for outcomes to occur?**		Partial	
**INTERNAL VALIDITY – HAS THIS STUDY BEEN CONDUCTED RIGOROUSLY IN ORDER TO REDUCE BIAS?**			
**SELECTION BIAS**	**Were the cases and controls taken from comparable populations?**	Yes	Cases and controls were women of reproductive age, recruited through public advertisement at the University Hospital.
	**Was the case definition adequate and established in a standard, valid and reliable way?**	Yes	Rotterdam criteria was used.
	**Was the control status established in a standard, valid and reliable way?**	No	No diagnostic criteria of PCOS were reported to have been performed on controls. However they report "A control group was set up with BMI-matched non-hirsute women with ovulatory cycles (mid-luteal progesterone 43.8 ng/ml)." which will not be sufficient to exclude PCOS in a control.

PERFORMANCE BIAS	**Aside from the exposure/ intervention, were the groups treated the same?**	Not reported	
	**Were outcome assessors blind to case and control status?**	Not reported	
	**Were all outcomes measured in a standard, valid and reliable way?**	Yes	The laboratory biochemical tests are standard, valid and reliable tests and the methods used are described.<br><br>Anthropometric measurements were performed in duplicate by two investigators. Skinfold thickness was estimated with a caliper (Cescorf, Mitutoyo). To estimate truncal adiposity, the sum of three skinfold measurements – subscapular, suprailiac, and abdominal was considered (referred to as 'sum of trunk skinfolds', expressed in mm).
	**Were outcomes assessed objectively and independently?**	Yes	The free androgen index was estimated using the formula T (nmol/l)/SHBG (nmol/l)x 100. HOMA was calculated by multiplying insulin (mIU/ml) by glucose (mmol/l) and dividing the product by 22.5.<br>The percentage of total body fat was calculated by the Faulkner formula: percent total body fat.(triceps.subscapular.suprailiac.abdominal skinfolds x 0.153)+5.783.
ATTRITION BIAS	**What percentage of the individuals recruited into each arm of the study were lost to follow up?**	Not reported	
	**What percentage of each group (cases and controls) refused to**	Not reported	

	**participate in the study?**		
	**What percentage of the individuals were not included in the analysis?**	Not reported	
**REPORT BIAS**	**Is the paper free of selective outcome reporting?**	Not reported	No published protocol available.
**CONFOUNDING**	**Were the groups comparable with regards to key prognostic variables?**	Partial	The groups were similar regarding BMI. Matching was done according to BMI. Age differed significantly at baseline.
**OTHER INTERNAL VALIDITY/BIAS**	**Were there any conflicts of interest in the writing or funding of this study?**	No	
	**Was the study sufficiently powered to detect any differences between the groups?**	Not reported	

Melbourne, Australia AND Wells G, Shea B, O'Connell D, Peterson J, Welch V, Losos M, Tugwell P. The Newcastle–Ottawa Scale (NOS) for assessing the quality of nonrandomized studies in meta-analyses. In: Proceedings of the 3rd Symposium on Systematic Reviews Beyond the Basics: Improving Quality and Impact. Oxford; 2000. p. 3-5).

Critical appraisal of a case control study

**If statistical analysis was undertaken, was this appropriate?**	Yes	"Results are presented as means+SD. Non-parametric data are presented as medians and interquartile range. Logarithmic transformation was performed for variables presenting non-normal distribution. Two-tailed Student t-tests were used to compare the means of two ... performed using the Statistical Package for the Social Sciences (SPSS, Chicago, IL). Data were considered to be significant at p<0.05. "
**Comments**	This study was designed as a single-blind randomized controlled study in four blocks. Women with PCOS and women without PCOS were randomized to either high protein diet or normal protein diet. In this review we compare the same treatment for two different groups, and therefore use the results for HP-group and NP-group separately.	
**What is the overall risk of bias?**	High	*High - Few or no criteria fulfilled or the conclusions of the study are likely or very likely to be affected.*

**Study ID**	Villa 1999	
**Study citation**	Villa, P., et al. (1999). "Effect of opioid blockade on insulin and growth hormone (GH) secretion in patients with polycystic ovary syndrome: the heterogeneity of impaired GH secretion is related to both obesity and hyperinsulinism." Fertility and sterility **71**(1): 115-121.	
**EXTERNAL VALIDITY – IS THIS STUDY AND ITS RESULTS GENERALIZABLE TO MY SYSTEMATIC REVIEW QUESTION?**		
**Patient/population/ participants**	Women with PCOS, age 26.3±5 years	
**Control population**	Women without PCOS, matched for BMI and age.	
**N**	The number of participants that were:<br>• Screened:-<br>• Enrolled: 36(22 PCOS 14 controls)<br>• Assessed: 36(22 PCOS 14 controls)<br>• Followed up:-	
**Setting**	Catholic University of Sacred Heart School of Medicine in Rome, Italy.	
**Intervention/indicator**	The patients underwent 4–5 weeks of outpatient treatment with 50 mg/d of naltrexone (Antaxone; Zamboh, Vicenza, Italy), an oral narcotic antagonist taken in the evening.	
**Outcomes**	• Fasting glucose<br>• LH (not relevant to systematic review)<br>• FSH (not relevant to systematic review)<br>• E2 (not relevant to systematic review)<br>• Testosterone (nmol/L)<br>• Androstenedione (not relevant to systematic review)<br>• DHEAS (not relevant to systematic review)<br>• 17-OHP (not relevant to systematic review)<br>• Insulin<br>• Growth hormone (not relevant to systematic review)<br>• BMI<br>• AUC of glucose (not relevant to systematic review)<br>• SHBG<br>• FAI	
**Does the study have a clearly focused question and/or PICO?**	Yes	
**Inclusion Criteria**	Yes	"Polycystic ovary syndrome was diagnosed by the presence of clinical findings (oligomenorrhea/amenorrhea and hirsutism),

<table>
<tr><td colspan="2"></td><td></td><td>echographic data (bilaterally normal or enlarged ovaries with increased stroma and at least 7–10 microcysts <5 mm in diameter at the time of transvaginal ultrasound examination), and elevated plasma androgen levels.”

Controls were healthy, normally ovulating volunteers.</td></tr>
<tr><td colspan="2">Exclusion Criteria</td><td>Yes</td><td>Adrenal enzymatic defects were excluded by an ACTH test, according to the criteria of New et al.</td></tr>
<tr><td colspan="2">If there were specified inclusion/ exclusion criteria, were these appropriate?</td><td>Partial</td><td>The authors have inclusion criteria, but don´t specify levels of androgens i.e. No inclusion criteria for controls are specified.</td></tr>
<tr><td colspan="2">Is a case control study the appropriate design to answer this question?</td><td>Yes</td><td></td></tr>
<tr><td colspan="2">Were the outcomes measured appropriate?</td><td>Yes</td><td></td></tr>
<tr><td colspan="2">Was there sufficient duration of follow-up for outcomes to occur?</td><td>Yes</td><td></td></tr>
<tr><td colspan="4">INTERNAL VALIDITY – HAS THIS STUDY BEEN CONDUCTED RIGOROUSLY IN ORDER TO REDUCE BIAS?</td></tr>
<tr><td rowspan="2">SELECTION BIAS</td><td>Were the cases and controls taken from comparable populations?</td><td>Not reported</td><td>Not reported where cases and controls were taken from, except that controls where healthy volunteers.</td></tr>
<tr><td>Was the case definition adequate and established in a standard, valid and reliable way?</td><td>Partial</td><td>Authors do not clearly describe the diagnostic criteria.</td></tr>
</table>

Cited in full as: Monash Centre for Health Research and Implementation (MCHRI) Evidence Synthesis Program template for critical appraisal of a case control study (2014),

	**Was the control status established in a standard, valid and reliable way?**	No	Controls are reported to be normally ovulation, but nothing is reported about hyperandrogenism or PCO. Therefore cannot be certain that controls are really controls and not cases.
PERFORMANCE BIAS	**Aside from the exposure/ intervention, were the groups treated the same?**	Not reported	
	**Were outcome assessors blind to case and control status?**	Not reported	
	**Were all outcomes measured in a standard, valid and reliable way?**	Yes	The laboratory biochemical tests are standard, valid and reliable tests and the methods used are described.
	**Were outcomes assessed objectively and independently?**	Yes	The free androgen index FAI was calculated with the use of the following ratio: testosterone x100/SHBG.<br>Body mass index (BMI) was calculated in each patient using the following formula: BMI =weight (kg)/height^2 (m^2).
ATTRITION BIAS	**What percentage of the individuals recruited into each arm of the study were lost to follow up?**	Not reported	
	**What percentage of each group (cases and controls) refused to participate in the study?**	Not reported	
	**What percentage of the individuals**	Not reported	

	**were not included in the analysis?**		
REPORT	**Is the paper free of selective outcome reporting?**	Not reported	There is no published protocol.
CONFOUNDING	**Were the groups comparable with regards to key prognostic variables?**	Yes	The groups were matched by age and BMI.
OTHER INTERNAL VALIDITY/BIAS	**Were there any conflicts of interest in the writing or funding of this study?**	Not reported	
	**Was the study sufficiently powered to detect any differences between the groups?**	Not reported	
	**If statistical analysis was undertaken, was this appropriate?**	Yes	"The distribution of the data was tested with the Kolmogorov-<br>Smirnov test to verify whether the samples came from a specified distribution, and we found that the data were not normally distributed. The significance of differences between the same tests performed before and after naltrexone treatment was assessed by the nonparametric Wilcoxon rank-sum test. The comparison between different study groups was performed by the nonparametric Mann-Whitney U -test. Linear regression analysis was used to analyze possible correlations between

			endocrine findings. The level of statistical significance was set at P <0.05."
**Comments**			
**What is the overall risk of bias?**		High	*High - Few or no criteria fulfilled or the conclusions of the study are likely or very likely to be affected.*

MCHRI – Monash University and Monash Health, Melbourne, Australia (*adapted from* Critical Appraisal Templates (2010) Centre for Clinical Effectiveness, Southern Health, Melbourne, Australia AND Wells G, Shea B, O'Connell D, Peterson J, Welch V, Losos M, Tugwell P. The Newcastle–Ottawa Scale (NOS) for assessing the quality of nonrandomized studies in meta-analyses. In: Proceedings of the 3rd Symposium on Systematic Reviews Beyond the Basics: Improving Quality and Impact. Oxford; 2000. p. 3-5).

Critical appraisal of a case control study

**Study ID**	Kahal 2015; Kahal 2013
**Study citation**	Kahal, H., et al. (2015). "The effects of treatment with liraglutide on atherothrombotic risk in obese young women with polycystic ovary syndrome and controls." BMC Endocrine Disorders **15**(14).<br><br>Kahal, H., et al. (2013). "Polycystic ovary syndrome has no independent effect on vascular, inflammatory or thrombotic markers when matched for obesity." Clinical Endocrinology **79**(2): 252–258.
**EXTERNAL VALIDITY – IS THIS STUDY AND ITS RESULTS GENERALIZABLE TO MY SYSTEMATIC REVIEW QUESTION?**	
**Patient/population/ participants**	Women with PCOS, age 33.9±6.7 years, BMI 37.9±5.0 kg/m^2
**Control population**	Women without PCOS, age 33.5±7.1 years, BMI 36.5±4.6

	kg/m²
**N**	The number of participants that were:<br>• Screened: 51<br>• Enrolled: 36 (19 PCOS, 17 non-PCOS)<br>• Assessed: 25 (13 PCOS, 12 non-PCOS)<br>• Followed up: -
**Setting**	UK
**Intervention/indicator**	"Liraglutide 0.6 mg od subcutaneous injection for 1 week, 1.2 mg od for one week and then 1.8 mg od thereafter for six months. Study participants received no dietary advice"
**Outcomes**	• Weight<br>• BMI<br>• Waist circumference (standard deviations not reported, data excluded)<br>• Blood pressure (standard deviations not reported, data excluded)<br>• Average heart rate (not relevant to systematic review)<br>• Metabolic syndrome (not relevant to systematic review)<br>• Testosterone (standard deviations not reported, data excluded)<br>• FAI (standard deviations not reported, data excluded)<br>• SHBG (standard deviations not reported, data excluded)<br>• Fasting plasma glucose (standard deviations not reported, data excluded)<br>• Fasting insulin (standard deviations not reported, data excluded)<br>• HOMA-IR (standard deviations not reported, data excluded)<br>• HOMA-β (not relevant to systematic review)<br>• High-sensitivity C-reactive protein (standard deviations not reported, data excluded)<br>• Total cholesterol (standard deviations not reported, data excluded)<br>• LDL (standard deviations not reported, data excluded)<br>• HDL (standard deviations not reported, data excluded)<br>• Triglycerides (standard deviations not reported, data excluded)<br>• Cholesterol/HDL ratio (not relevant to systematic review)

	• Isoprostane (not relevant to systematic review)<br>• Fibrinogen (standard deviations not reported, data excluded)<br>• Carotid intima-media wall thickness (not relevant to systematic review)<br>• Endotheial function measurements (not relevant to systematic review)<br>• Platelet function, activation and sensitivity measurements (not relevant to systematic review)<br>• Clot function/lysis measurements (not relevant to systematic review)	
**Does the study have a clearly focused question and/or PICO?**	Yes	
**Inclusion Criteria**	Yes	Women with or without PCOS, "BMI between 30–45 kg/m^2, were between 18–45 years of age and were not taking the oral contraceptive pill or any medications that might influence study results including metformin"
**Exclusion Criteria**	Yes	"Other endocrine disorders with similar presentation were excluded including hypothyroidism, hyperprolactinaemia, Cushing's disease, hypopituitarism, androgen producing tumours and non-classic congenital adrenal hyperplasia". For the controls, those "with a history of clinical or biochemical hirsutism or menstrual irregularities were excluded…"Participants with an alcohol intake of >14 units/week were also excluded"
**If there were specified inclusion/ exclusion criteria, were these appropriate?**	Yes	
**Is a case control study the appropriate design to answer this question?**	Yes	
**Were the outcomes measured appropriate?**	Yes	

Cited in full as: Monash Centre for Health Research and Implementation (MCHRI) Evidence Synthesis Program template for critical appraisal of a case control study (2014),

**Was there sufficient duration of follow-up for outcomes to occur?**		Yes	
**INTERNAL VALIDITY – HAS THIS STUDY BEEN CONDUCTED RIGOROUSLY IN ORDER TO REDUCE BIAS?**			
**SELECTION BIAS**	**Were the cases and controls taken from comparable populations?**	Yes	
	**Was the case definition adequate and established in a standard, valid and reliable way?**	Yes	Rotterdam criteria used to diagnose PCOS
	**Was the control status established in a standard, valid and reliable way?**	Yes	
**PERFORMANC E BIAS**	**Aside from the exposure/ intervention, were the groups treated the same?**	Yes	
	**Were outcome assessors blind to case and control status?**	Not reported	Not reported for all outcomes of interest

	**Were all outcomes measured in a standard, valid and reliable way?**	Not reported	No description of protocol for anthropometric measurements
	**Were outcomes assessed objectively and independently?**	Not reported	Not reported for all outcomes of interest
**ATTRITION BIAS**	**What percentage of the individuals recruited into each arm of the study were lost to follow up?**	Not reported	Only reported overall, not for each group
	**What percentage of each group (cases and controls) refused to participate in the study?**	Not reported	
	**What percentage of the individuals were not included in the analysis?**	X% treatment<br>X% control/ comparison	PCOS= 32%<br>Non-PCOS= 29%
**REPORT**	**Is the paper free of selective outcome reporting?**	No	Depression and quality of life not reported (as per clinical trial reg. no. ISRCTN48560305)
**CONFOUNDING**	**Were the groups comparable with regards to key prognostic variables?**	Yes	

MCHRI – Monash University and Monash Health, Melbourne, Australia (*adapted from* Critical Appraisal Templates (2010) Centre for Clinical Effectiveness, Southern Health, Melbourne, Australia AND Wells G, Shea B, O'Connell D, Peterson J, Welch V, Losos M, Tugwell P. The Newcastle–Ottawa Scale (NOS) for assessing the quality of nonrandomized studies in meta-analyses. In: Proceedings of the 3rd Symposium on Systematic Reviews Beyond the Basics: Improving Quality and Impact. Oxford; 2000. p. 3-5).

OTHER INTERNAL VALIDITY/BIAS			
OTHER INTERNAL VALIDITY/BIAS	**Were there any conflicts of interest in the writing or funding of this study?**	No	
	**Was the study sufficiently powered to detect any differences between the groups?**	No	Required sample size of 18 participants per group to detect "an effect size of 1.12 (or larger) with 80% power, 20% attrition and 5% significance (two-tailed) between cases and controls" was not retained.
	**If statistical analysis was undertaken, was this appropriate?**	Yes	
**Comments**			
**What is the overall risk of bias?**		High	*High - Few or no criteria fulfilled or the conclusions of the study are likely or very likely to be affected.*

Critical appraisal of a case control study

**Study ID**	Nikokavoura 2015
**Study citation**	Nikokavoura, E. A., et al. (2015). "Weight loss for women with and without polycystic ovary syndrome following a very low-calorie diet in a community-based setting with trained facilitators for 12 weeks." Diabetes, Metabolic Syndrome and Obesity: Targets and Therapy **8**: 495-503.
**EXTERNAL VALIDITY – IS THIS STUDY AND ITS RESULTS GENERALIZABLE TO MY SYSTEMATIC REVIEW QUESTION?**	

**Patient/population/ participants**	Women with PCOS, age 35.7±8.9 years, BMI 40.0±6.3 kg/m2	
**Control population**	Women without PCOS, age 35.8±8.9 years, BMI 40.0±6.3 kg/m2	
**N**	The number of participants that were:<br>• Screened: 102,610 LighterLife participants<br>• Included in study: 1016 (508 PCOS, 508 control)<br>• Assessed as part of completers analysis: 274 (137 PCOS, 137 control)<br>• Followed up: -	
**Setting**	UK	
**Intervention/indicator**	"The intervention used was a commercial weight-management program (LighterLife Total)... The VLCD provides an average daily intake of 600 kcal (50 g protein, 50 g carbohydrate, mean 17 g fat, ie, 36% energy from protein, 36% carbohydrate, and 28% fat) in the form of food packs (soups, shakes, textured meals, and bars) that contain ≥100% recommended daily allowances for vitamins and minerals, including vitamins A, C, D, E, K, thiamine, riboflavin, niacin, B6 , B12, folic acid, biotin, pantothenic acid, calcium, phosphorous, iron, zinc, magnesium, iodine, potassium, sodium, copper, manganese, selenium, molybdenum, chromium, chloride, and fluoride. Clients were also able to purchase an ancillary "fiber mix" to add to their water, which contained inulin as the source of fiber. Participants undertook the VLCD alongside a unique behavior-change program developed specifically for weight management in the obese. This is informed by concepts from cognitive behavioral therapy and transactional analysis (transactional cognitive behavioral therapy) and addiction/ change theory. It is delivered in small, single-sex, weekly groups by weight-management counselors who are specifically trained in the facilitation of behavior change for the treatment of obesity"	
**Outcomes**	• Weight<br>• BMI<br>• Blood pressure (only reported for baseline observation carried forward data, not for completers analysis)	
**Does the study have a clearly focused question and/or PICO?**	Yes	

**Inclusion Criteria**	Yes	Women with or without PCOS who had self-referred onto the VLCD program
**Exclusion Criteria**	Yes	" Participants were excluded from taking part in the LighterLife Total program, and consequently from the study as well, if they met any of the following criteria: type 1 diabetes; porphyria; total lactose intolerance; major cardiovascular or cerebrovascular disease; history of renal disorder or hepatic disease; active cancer; epilepsy, seizures, convulsions, major depressive disorder, psychotic episodes, schizophrenia, bipolar disorders, or delusional disorders; current suffering from anorexia, bulimia, or undergoing treatment for any other eating disorder; pregnant or breastfeeding; had given birth or had a miscarriage in the last 3 months"
**If there were specified inclusion/ exclusion criteria, were these appropriate?**	Yes	
**Is a case control study the appropriate design to answer this question?**	Yes	
**Were the outcomes measured appropriate?**	Yes	
**Was there sufficient duration of follow-up for outcomes to occur?**	Yes	
**INTERNAL VALIDITY – HAS THIS STUDY BEEN CONDUCTED RIGOROUSLY IN ORDER TO REDUCE BIAS?**		

Cited in full as: Monash Centre for Health Research and Implementation (MCHRI) Evidence Synthesis Program template for critical appraisal of a cohort study (2014), MCHRI

SELECTION BIAS	**Were the cases and controls taken from comparable populations?**	Yes	
	**Was the case definition adequate and established in a standard, valid and reliable way?**	Not reported	PCOS diagnosis criteria not reported.
	**Was the control status established in a standard, valid and reliable way?**	Yes	
PERFORMANC E BIAS	**Aside from the exposure/ intervention, were the groups treated the same?**	Yes	
	**Were outcome assessors blind to case and control status?**	Not reported	
	**Were all outcomes measured in a standard, valid and reliable way?**	Not reported	"Measurements of height and weight took place in LighterLife centers, and were carried out by facilitators who had been trained and provided with protocols developed within LighterLife. The actual types of equipment used for these measurements were not recorded. Measurements were taken during weekly meetings that occurred at the same location and time each week". Insufficient information provided to determine whether measurements were valid.
	**Were outcomes assessed**	Not reported	

	**objectively and independently?**		
ATTRITION BIAS	**What percentage of the individuals recruited into each arm of the study were lost to follow up?**	Not reported	
	**What percentage of each group (cases and controls) refused to participate in the study?**	Not reported	
	**What percentage of the individuals were not included in the analysis?**	X% treatment<br>X% control/ comparison	PCOS= 73%<br>Non-PCOS= 73%<br>For the completers analysis. Were included in the baseline observation carried forward analysis.
REPORT BIAS	**Is the paper free of selective outcome reporting?**	Not reported	No report of protocol registration.
CONFOUNDING	**Were the groups comparable with regards to key prognostic variables?**	Yes	
OTHER INTERNAL VALIDITY/BIAS	**Were there any conflicts of interest in the writing or funding of this study?**	Yes	"CR has received lecture honoraria and has attended national/ international meetings as a guest of LighterLife UK. EAN has received funding and has attended national/international meetings as a guest of LighterLife UK. CR, JB, and WLW have been involved with other companies with an interest in obesity. KLJ is employed by LighterLife UK. JB was an adviser to LighterLife UK"

– Monash University and Monash Health, Melbourne, Australia (*adapted from* Critical Appraisal Templates (2010) Centre for Clinical Effectiveness, Southern Health, Melbourne, Australia AND Wells G, Shea B, O'Connell D, Peterson J, Welch V, Losos M, Tugwell P. The

	**Was the study sufficiently powered to detect any differences between the groups?**	No	Sample size required not retained for completers analysis
	**If statistical analysis was undertaken, was this appropriate?**	Yes	
**Comments**		Data reported here is for the completers analysis only.	
**What is the overall risk of bias?**		High	*High - Few or no criteria fulfilled or the conclusions of the study are likely or very likely to be affected.*

Newcastle–Ottawa Scale (NOS) for assessing the quality of nonrandomized studies in meta-analyses. In: Proceedings of the 3rd Symposium on Systematic Reviews Beyond the Basics: Improving Quality and Impact. Oxford; 2000. p. 3-5).

Critical appraisal of a case control study

**Study ID**	Cheang 2016
**Study citation**	Cheang K.I., et al. (2016). "Effect on Insulin-Stimulated Release of D-Chiro-Inositol-Containing Inositolphosphoglycan Mediator during Weight Loss in Obese Women with and without Polycystic Ovary Syndrome". International Journal of Endocrinology **2016**: no pagination.
**EXTERNAL VALIDITY – IS THIS STUDY AND ITS RESULTS GENERALIZABLE TO MY SYSTEMATIC REVIEW QUESTION?**	
**Patient/population/ participants**	Women with PCOS, age 26.9±4.6 years, BMI 36.6±5.1 kg/m²
**Control population**	Women without PCOS, age 27.5±5.7 years, BMI 35.8±4.8 kg/m²
**N**	• Screened: 80 provided consent to participate<br>• Enrolled: 61 (34 PCOS, 27 non-PCOS)

	• Assessed: 31 (16 PCOS, 15 non-PCOS)<br>• Followed up: -	
**Setting**	Clinical Research Service Unit of Virginia Commonwealth University's Center for Clinical and Translational Research, USA	
**Intervention/indicator**	"The women were instructed to follow an 8-week course of standardized hypocaloric diet containing 50% carbohydrates, 30% total lipids, and 20% proteins. They were instructed to maintain these hypocaloric diets by caloric restriction to create a deficit of 500–1000 kcal/day, as per obesity management guidelines of the National Heart, Lung, and Blood Institute…. The women were instructed specifically to avoid making any conscious effort to modify physical activity or attempt other weight loss methods in addition to the hypocaloric diets per this protocol…. During this 8-week period, the participants purchased and prepared their own meals and maintained daily food logs"	
**Outcomes**	• Weight<br>• BMI<br>• Waist to hip ratio<br>• Fasting insulin<br>• Fasting glucose<br>• AUC glucose (not relevant to systematic review)<br>• AUC insulin (not relevant to systematic review)<br>• Matsuda index (not relevant to systematic review)<br>• AUC DCI-IPG (not relevant to systematic review)<br>• Ratio of AUC DCI-IPG/AUC insulin (not relevant to systematic review)	
**Does the study have a clearly focused question and/or PICO?**	Yes	
**Inclusion Criteria**	Yes	"Obese (≥30 kg/m²) and between the ages of 18 and 40 years. PCOS was defined by the modified Rotterdam criteria, after excluding other endocrine disorders… The control group consisted of regular cycling women with normal serum testosterone"
**Exclusion Criteria**	Yes	"The exclusion criteria for all women included weight loss attempts by either diet or exercise within 3 months of study participation, diabetes mellitus by fasting glucose or oral glucose tolerance test (OGTT),

		clinically significant pulmonary, cardiac, renal, hepatic, neurologic, psychiatric, infectious, neoplastic, and malignant disease, or pregnancy as documented by urine hCG. PCOS women with disorders associated with insulin resistance, for example, hypertension or dyslipidemia, were not excluded as long as they had been on a stable dose of medication for 6 months. Normal women were excluded if they had a history of gestational diabetes or had a first-degree relative with diabetes or if they demonstrated abnormal glucose tolerance at baseline or if they had hypertension or dyslipidemia”
**If there were specified inclusion/ exclusion criteria, were these appropriate?**	Yes	
**Is a case control study the appropriate design to answer this question?**	Yes	
**Were the outcomes measured appropriate?**	Yes	
**Was there sufficient duration of follow-up for outcomes to occur?**	Yes	
**INTERNAL VALIDITY – HAS THIS STUDY BEEN CONDUCTED RIGOROUSLY IN ORDER TO REDUCE BIAS?**		
**SELECTION BIAS** – **Were the cases and controls taken from comparable populations?**	Not reported	

	**Was the case definition adequate and established in a standard, valid and reliable way?**	Yes	PCOS diagnosed according to modified Rotterdam criteria
	**Was the control status established in a standard, valid and reliable way?**	Yes	
PERFORMANCE BIAS	**Aside from the exposure/ intervention, were the groups treated the same?**	Yes	
	**Were outcome assessors blind to case and control status?**	Not reported	
	**Were all outcomes measured in a standard, valid and reliable way?**	Partial	"Height and weight were measured to the nearest 0.1 cm and 0.1 kg using a precision stadiometer and digital scale. Waist was measured at the level of the umbilicus, and hip circumference was measured at the widest diameter of the buttocks to the nearest 0.1 cm". Whilst weight was reported to be measured in the fasting state, not reported whether the participants were clothed or not. Additionally, measuring waist circumference at the level of the umbilicus may be an underestimate of the true waist circumference.
	**Were outcomes assessed objectively and independently?**	Partial	
ATTRITI	**What percentage of the individuals**	X% treatment	PCOS group= 53%<br>Control group= 44%

	**recruited into each arm of the study were lost to follow up?**	X% control/ comparison	Reasons for drop out not reported.
	**What percentage of each group (cases and controls) refused to participate in the study?**	Not reported	
	**What percentage of the individuals were not included in the analysis?**	X% treatment<br>X% control/ comparison	PCOS group= 53%<br>Control group= 44%
**REPORT BIAS**	**Is the paper free of selective outcome reporting?**	Not reported	No report of protocol registration
**CONFOUNDING**	**Were the groups comparable with regards to key prognostic variables?**	Yes	"At baseline, control women and women with PCOS did not differ in terms of age, racial mix, BMI, or waist-to-hip ratio"
**OTHER INTERNAL VALIDITY/BIAS**	**Were there any conflicts of interest in the writing or funding of this study?**	No	"The authors declare that they have no competing interests"
	**Was the study sufficiently powered to detect any differences between the groups?**	Not reported	No report of a power calculation

	**If statistical analysis was undertaken, was this appropriate?**	Yes	
**Comments**			
**What is the overall risk of bias?**		Moderate	*Moderate - Some of the criteria have been fulfilled and those criteria that have not been fulfilled may affect the conclusions of the study.*

Cited in full as: Monash Centre for Health Research and Implementation (MCHRI) Evidence Synthesis Program template for critical appraisal of a cohort study (2014), MCHRI – Monash University and Monash Health, Melbourne, Australia (*adapted from* Critical Appraisal Templates (2010) Centre for Clinical Effectiveness, Southern Health, Melbourne, Australia AND Wells G, Shea B, O'Connell D, Peterson J, Welch V, Losos M, Tugwell P. The Newcastle–Ottawa Scale (NOS) for assessing the quality of nonrandomized studies in meta-analyses. In: Proceedings of the 3rd Symposium on Systematic Reviews Beyond the Basics: Improving Quality and Impact. Oxford; 2000. p. 3-5).

Critical appraisal of a case control study

**Study ID**	Kogure 2016
**Study citation**	Kogure, G. S., et al. (2016). "Resistance Exercise Impacts Lean Muscle Mass in Women with Polycystic Ovary Syndrome". Medicine & Science in Sports & Exercise **48**(4): 589-598.
**EXTERNAL VALIDITY – IS THIS STUDY AND ITS RESULTS GENERALIZABLE TO MY SYSTEMATIC REVIEW QUESTION?**	
**Patient/population/ participants**	Women with PCOS, age 28.1±5.4 years, BMI 28.4±6.0 kg/m²
**Control population**	Women without PCOS, age 29.6±5.2 years, BMI 26.2±5.7 kg/m²
**N**	The number of participants that were:<br>• Screened: 196 (85 PCOS, 111 control)<br>• Included : 170 (73 PCOS, 97 control)-agreed to participate

	• Assessed: 97 (45 PCOS, 52 control)<br>• Followed up: -
**Setting**	Progressive resistance training was performed at the Centre of Physical Education, Recreation, and Sports at the University of São Paulo, Brazil
**Intervention/indicator**	"The volunteers received training for familiarization with the exercises and underwent an adaptation period of 2 wk or six adaptation sets; the initial load intensity of all exercises after the adaptation period was based on dynamic maximum muscle strength based on the one-repetition maximum test… Physical education professionals supervised each exercise, and a linear periodization of training was prepared that followed a trend of decreasing volume and increasing intensity throughout the training period. In this training protocol, the exercise intensity was increased in each microcycle, whereas the number of repetitions was decreased (maintaining a minimum of eight repetitions) because of increased overload. The exercises included bench presses, leg extensions, front lat pull-downs, leg curls, lateral raises, leg presses, triceps pulleys, calf leg presses, arm curls, and abdominal exercises executed in alternating segments. The training duration program for each participant was approximately 1 hour/day three times a week for 4 months…We provided a Centre of Physical Education, Recreation, and Sports membership, light meal after each PRT session, and a pair of running shoes to increase participant compliance and adherence… The subjects were instructed not to undertake any regular or supervised exercise during the PRT duration"
**Outcomes**	• Weight<br>• BMI<br>• Waist circumference<br>• LH (not relevant to systematic review)<br>• FSH (not relevant to systematic review)<br>• Oestradiol (not relevant to systematic review)<br>• Androstenedione (not relevant to systematic review)<br>• Total testosterone<br>• SHBG<br>• FAI<br>• Fasting glucose<br>• Fasting insulin<br>• HOMA-IR<br>• Total lean muscle mass

	• Trunk lean muscle mass (not relevant to systematic review)<br>• Muscle mass index (not relevant to systematic review)<br>• Appendicular lean mass (not relevant to systematic review)<br>• % body fat<br>• Menstrual history (only for PCOS group, incomplete data reported)	
**Does the study have a clearly focused question and/or PICO?**	Yes	
**Inclusion Criteria**	Yes	Women with or without PCOS. "18–37 yr of age, any race, any social status, sedentary or did not engage in regular supervised physical activity, and BMI indicating a normal weight status (18–25 kg/m²) or an overweight status (25–29.0 kg/m²) or first-degree obesity (>30 kg/m²) according to the World Health Organization criteria"
**Exclusion Criteria**	Yes	"The presence of systemic diseases, hormonal contraceptive use, smoking, and pregnancy"
**If there were specified inclusion/ exclusion criteria, were these appropriate?**	Yes	
**Is a case control study the appropriate design to answer this question?**	Yes	
**Were the outcomes measured appropriate?**	Yes	
**Was there sufficient duration of follow-up for outcomes to occur?**	Yes	

INTERNAL VALIDITY – HAS THIS STUDY BEEN CONDUCTED RIGOROUSLY IN ORDER TO REDUCE BIAS?			
SELECTION BIAS	**Were the cases and controls taken from comparable populations?**	Yes	
	**Was the case definition adequate and established in a standard, valid and reliable way?**	Yes	Rotterdam criteria
	**Was the control status established in a standard, valid and reliable way?**	Yes	
PERFORMANCE BIAS	**Aside from the exposure/ intervention, were the groups treated the same?**	Yes	
	**Were outcome assessors blind to case and control status?**	Not reported	
	**Were all outcomes measured in a standard, valid and reliable way?**	Yes	
	**Were outcomes assessed objectively and independently?**	Yes	
ATT RITI	**What percentage of the individuals**	X% treatment	PCOS= 3%<br>Non-PCOS= 2%

	**recruited into each arm of the study were lost to follow up?**	X% control/ comparison	
	**What percentage of each group (cases and controls) refused to participate in the study?**	Not reported	
	**What percentage of the individuals were not included in the analysis?**	X% treatment<br>X% control/ comparison	PCOS= 38%<br>Non-PCOS= 46%
**REPORT BIAS**	**Is the paper free of selective outcome reporting?**	Yes	
**CONFOUNDING**	**Were the groups comparable with regards to key prognostic variables?**	Yes	
**OTHER INTERNAL VALIDITY/BIAS**	**Were there any conflicts of interest in the writing or funding of this study?**	No	
	**Was the study sufficiently powered to detect any differences between the groups?**	No	Required sample size of 60 participants per group to "observe a 10% mean difference between groups, have 80% statistical power, and a significance level of 0.05" were not retained until end of intervention

	**If statistical analysis was undertaken, was this appropriate?**	Yes	
**Comments**			
**What is the overall risk of bias?**		Moderate	*Moderate - Some of the criteria have been fulfilled and those criteria that have not been fulfilled may affect the conclusions of the study.*

Cited in full as: Monash Centre for Health Research and Implementation (MCHRI) Evidence Synthesis Program template for critical appraisal of a cohort study (2014), MCHRI – Monash University and Monash Health, Melbourne, Australia (*adapted from* Critical Appraisal Templates (2010) Centre for Clinical Effectiveness, Southern Health, Melbourne, Australia AND Wells G, Shea B, O'Connell D, Peterson J, Welch V, Losos M, Tugwell P. The Newcastle–Ottawa Scale (NOS) for assessing the quality of nonrandomized studies in meta-analyses. In: Proceedings of the 3rd Symposium on Systematic Reviews Beyond the Basics: Improving Quality and Impact. Oxford; 2000. p. 3-5).

Critical appraisal of a case control study

**Study ID**	Bhandari 2016
**Study citation**	Bhandari, S., et al. (2016). "Effect of sleeve gastrectomy bariatric surgery-induced weight loss on serum AMH levels in reproductive aged women." Gynecological Endocrinology **32**(10): 799-802.
**EXTERNAL VALIDITY – IS THIS STUDY AND ITS RESULTS GENERALIZABLE TO MY SYSTEMATIC REVIEW QUESTION?**	
**Patient/population/ participants**	Women with PCOS, age 27.77±4.50 years, BMI 42.52±5.71 kg/m²
**Control population**	Women without PCOS, age 29.34±4.96 years, BMI 45.03±6.11 kg/m²
**N**	The number of participants that were:<br>• Screened: not reported<br>• Enrolled: not reported<br>• Assessed: 75 (43 PCOS, 32 non-PCOS)<br>• Followed up: -
**Setting**	Department of Reproductive Medicine, of a tertiary care

	hospital, India	
**Intervention/indicator**	Sleeve gastrectomy (bariatric surgery)	
**Outcomes**	• Weight<br>• BMI<br>• Serum AMH (not relevant to systematic review)<br>• Abnormal cycles	
**Does the study have a clearly focused question and/or PICO?**	Yes	
**Inclusion Criteria**	Yes	"Females aged between 20 and 35 years with BMI >35 kg/m² undergoing sleeve gastrectomy (bariatric surgery) were considered for the study". PCOS and non-PCOS women.
**Exclusion Criteria**	Yes	"Potential participants were excluded if they were using any<br>hormonal treatment or fertility drugs like oral contraceptive pills<br>and/or metformin, had systemic diseases like hypothyroidism or hyperprolactinaemia. Patients who did not report for follow up, or had any surgical complication intra-operatively or post operatively<br>were also excluded from the study"
**If there were specified inclusion/ exclusion criteria, were these appropriate?**	Yes	
**Is a case control study the appropriate design to answer this question?**	Yes	
**Were the outcomes measured appropriate?**	Yes	
**Was there sufficient duration of follow-up for outcomes to occur?**	Yes	

**INTERNAL VALIDITY – HAS THIS STUDY BEEN CONDUCTED RIGOROUSLY IN ORDER TO REDUCE BIAS?**			
**SELECTION BIAS**	**Were the cases and controls taken from comparable populations?**	Yes	
	**Was the case definition adequate and established in a standard, valid and reliable way?**	Yes	“PCOS was diagnosed on basis of current Rotterdam criteria: 1) oligo- or anovulation, 2) clinical and/or biochemical signs of hyperandrogenism, or 3) polycystic ovaries (patient is diagnosed as PCOS if two or more features are present). Patient was classified as hyperandrogenism if total serum testosterone level480 ng/dL.”
	**Was the control status established in a standard, valid and reliable way?**	Yes	
**PERFORMANC E BIAS**	**Aside from the exposure/ intervention, were the groups treated the same?**	Yes	
	**Were outcome assessors blind to case and control status?**	Not reported	
	**Were all outcomes measured in a standard, valid and reliable way?**	Not reported	Protocol for anthropometric measurements not reported.
	**Were outcomes assessed objectively and independently?**	Not reported	

ATTRITION BIAS	**What percentage of the individuals recruited into each arm of the study were lost to follow up?**	Not reported	
	**What percentage of each group (cases and controls) refused to participate in the study?**	Not reported	
	**What percentage of the individuals were not included in the analysis?**	Not reported	
REPORT BIAS	**Is the paper free of selective outcome reporting?**	Not reported	No report of protocol registration
CONFOUNDING	**Were the groups comparable with regards to key prognostic variables?**	Yes	
OTHER INTERNAL VALIDITY/BIAS	**Were there any conflicts of interest in the writing or funding of this study?**	No	

Cited in full as: Monash Centre for Health Research and Implementation (MCHRI) Evidence Synthesis Program template for critical appraisal of a cohort study (2014), MCHRI – Monash University and Monash Health, Melbourne, Australia (*adapted from* Critical Appraisal Templates (2010) Centre for Clinical Effectiveness, Southern Health, Melbourne, Australia AND Wells G, Shea B, O'Connell D, Peterson J, Welch V, Losos M, Tugwell P. The Newcastle–Ottawa Scale (NOS) for assessing the quality of nonrandomized studies in

	**Was the study sufficiently powered to detect any differences between the groups?**	Not reported	No report of a power calculation
	**If statistical analysis was undertaken, was this appropriate?**	Yes	
**Comments**			
**What is the overall risk of bias?**		High	*High - Few or no criteria fulfilled or the conclusions of the study are likely or very likely to be affected.*

meta-analyses. In: Proceedings of the 3rd Symposium on Systematic Reviews Beyond the Basics: Improving Quality and Impact. Oxford; 2000. p. 3-5).

Critical appraisal of a case control study

**Study ID**	Al-Eisa 2017
**Study citation**	Al-Eisa, E., et al (2017). "Effects of supervised aerobic training on the levels of anti-mullerian hormone and adiposity measures in women with normo-ovulatory and polycystic ovary syndrome". Journal of the Pakistan Medical Association **67**(4): 499-507.
**EXTERNAL VALIDITY – IS THIS STUDY AND ITS RESULTS GENERALIZABLE TO MY SYSTEMATIC REVIEW QUESTION?**	
**Patient/population/ participants**	Women with PCOS, age 27.9 ± 4.1 years, BMI 33.45±2.75 kg/m^2
**Control population**	Women without PCOS, age 27.6 ± 5.7 years, BMI 31.7± 3.8 kg/m^2

**N**	The number of participants that were:<br>• Screened: not reported<br>• Enrolled: not reported<br>• Assessed: 60 (30 PCOS, 30 obese non-PCOS)<br>• Followed up: -
**Setting**	Obstetrics and Gynecology clinic, Mansoura University Hospital, Faculty of Medicine, Mansoura, Egypt
**Intervention/indicator**	"Training interventions programme of treadmill walking, 45 minutes three times per week for 12 weeks. Each individual's training intensity was calculated as the training heart rate (THR) based on the subject's age and predicted maximum heart rate and resting heart rate according to Karvonen's formula, [THR = resting heart rate (HRrest) + (maximum heart rate (HRmax) - HRrest) × training fraction (TF)], where TF was 65% to 75% for the moderate intensity used in this study. Each exercise session consisted of three phases, i.e. warm-up, active and cool-down phases. The cool-down phase continued for 10 to 15 minutes during which the workload gradually decreased until HR and blood pressure (BP) nearly returned to their resting levels. Throughout the training session, the subjects were monitored by a portable heart rate monitor to keep the exercise intensity within the pre-calculated training heart rate for each subject"
**Outcomes**	• BMI<br>• Weight<br>• Waist circumference<br>• Waist to hip ratio<br>• Ferriman-Gallwey score<br>• *Follicle-stimulating hormone (not relevant to systematic review)*<br>• Oestrogen *(not relevant to systematic review)*<br>• Prolactin *(not relevant to systematic review)*<br>• Adiponectin *(not relevant to systematic review)*<br>• Anti-Mullerian hormone *(not relevant to systematic review)*<br>• *Fasting glucose*<br>• *Fasting insulin*<br>• Fasting glucose-to-insulin ratio<br>• HOMA-IR<br>• Antral follicle count (not relevant to systematic review)<br>• Reproductive outcomes (not reported for all participants, thus not included in systematic review)

**Does the study have a clearly focused question and/or PICO?**	Yes	
**Inclusion Criteria**	Yes	"Obese women with or without polycystic ovary syndrome aged between 20 and 35 years referred to Obstetrics and Gynecology clinic, Mansoura University Hospital, Faculty of Medicine, Mansoura, Egypt"
**Exclusion Criteria**	Yes	"Participants with normal BMI, other concomitant diseases such as diabetes, viral infections, current and previous drug administration that affected hormonal levels were excluded"
**If there were specified inclusion/ exclusion criteria, were these appropriate?**	Yes	
**Is a case control study the appropriate design to answer this question?**	Yes	
**Were the outcomes measured appropriate?**	Yes	
**Was there sufficient duration of follow-up for outcomes to occur?**	Yes	
**INTERNAL VALIDITY – HAS THIS STUDY BEEN CONDUCTED RIGOROUSLY IN ORDER TO REDUCE BIAS?**		
**SELECTION BIAS** — **Were the cases and controls taken from comparable populations?**	Yes	

	**Was the case definition adequate and established in a standard, valid and reliable way?**	Yes	“Diagnosis of PCOS was established according to Ferriman-Gallwey scores. Based on the presence of elevated serum fertility hormones, disorder in biochemical hyper-androgenism, presence of chronic anovulation and Ferriman score of >8. Women with an ovulatory infertility were diagnosed according to irregular periods, normal fertility hormones and regular sexual intercourse.”<br>Criteria aligns with Rotterdam criteria.
	**Was the control status established in a standard, valid and reliable way?**	Yes	
**PERFORMANC E BIAS**	**Aside from the exposure/ intervention, were the groups treated the same?**	Yes	
	**Were outcome assessors blind to case and control status?**	Not reported	
	**Were all outcomes measured in a standard, valid and reliable way?**	Not reported	Protocol for anthropometric measurements not reported, not for the Ferriman-Gallwey score
	**Were outcomes assessed objectively and independently?**	Not reported	
**ATTRITION BIAS**	**What percentage of the individuals recruited into each arm of the study were lost to follow up?**	Not reported	

	**What percentage of each group (cases and controls) refused to participate in the study?**	Not reported	
	**What percentage of the individuals were not included in the analysis?**	Not reported	
**REPORT BIAS**	**Is the paper free of selective outcome reporting?**	Not reported	No report of a study protocol being registered
**CONFOUNDING**	**Were the groups comparable with regards to key prognostic variables?**	Not reported	Between-group comparisons not conducted at baseline
**OTHER INTERNAL VALIDITY/BIAS**	**Were there any conflicts of interest in the writing or funding of this study?**	No	
	**Was the study sufficiently powered to detect any differences between the groups?**	Not reported	No report of a power calculation

Cited in full as: Monash Centre for Health Research and Implementation (MCHRI) Evidence Synthesis Program template for critical appraisal of a cohort study (2014), MCHRI – Monash University and Monash Health, Melbourne, Australia (*adapted from* Critical Appraisal Templates (2010) Centre for Clinical Effectiveness, Southern Health, Melbourne, Australia AND Wells G, Shea B, O'Connell D, Peterson J, Welch V, Losos M, Tugwell P. The Newcastle–Ottawa Scale (NOS) for assessing the quality of nonrandomized studies in

	**If statistical analysis was undertaken, was this appropriate?**	Yes	
**Comments**			
**What is the overall risk of bias?**		High	*High - Few or no criteria fulfilled or the conclusions of the study are likely or very likely to be affected.*

meta-analyses. In: Proceedings of the 3rd Symposium on Systematic Reviews Beyond the Basics: Improving Quality and Impact. Oxford; 2000. p. 3-5).
